# Drug Nanocrystals: Focus on Brain Delivery from Therapeutic to Diagnostic Applications

**DOI:** 10.3390/pharmaceutics14040691

**Published:** 2022-03-23

**Authors:** Elide Zingale, Angela Bonaccorso, Claudia Carbone, Teresa Musumeci, Rosario Pignatello

**Affiliations:** 1 Laboratory of Drug Delivery Technology, Department of Drug and Health Sciences, University of Catania, Viale A. Doria 6, 95125 Catania, Italy; elidezingale@gmail.com (E.Z.); ccarbone@unict.it (C.C.); tmusumec@unict.it (T.M.); r.pignatello@unict.it (R.P.); 2 NANO-i—Research Centre on Ocular Nanotechnology, University of Catania, 95125 Catania, Italy

**Keywords:** nanomedicine, amorphism, central nervous system, solubility, nanotechnology

## Abstract

The development of new drugs is often hindered by low solubility in water, a problem common to nearly 90% of natural and/or synthetic molecules in the discovery pipeline. Nanocrystalline drug technology involves the reduction in the bulk particle size down to the nanosize range, thus modifying its physico-chemical properties with beneficial effects on drug bioavailability. Nanocrystals (NCs) are carrier-free drug particles surrounded by a stabilizer and suspended in an aqueous medium. Due to high drug loading, NCs maintain a potent therapeutic concentration to produce desirable pharmacological action, particularly useful in the treatment of central nervous system (CNS) diseases. In addition to the therapeutic purpose, NC technology can be applied for diagnostic scope. This review aims to provide an overview of NC application by different administration routes, especially focusing on brain targeting, and with a particular attention to therapeutic and diagnostic fields. NC therapeutic applications are analyzed for the most common CNS pathologies (i.e., Parkinson’s disease, psychosis, Alzheimer’s disease, etc.). Recently, a growing interest has emerged from the use of colloidal fluorescent NCs for brain diagnostics. Therefore, the use of NCs in the imaging of brain vessels and tumor cells is also discussed. Finally, the clinical effectiveness of NCs is leading to an increasing number of FDA-approved products, among which the NCs approved for neurological disorders have increased.

## 1. Introduction

The development of new drugs is often hindered by low solubility in water, a problem associated with nearly 90% of molecules in the discovery pipeline [1]. According to the literature, an increasing number of active pharmaceutical ingredients (APIs) of natural or synthetic origin show this behavior. The term “poor solubility” is applied when the maximum concentration of drug dissolved in water is <10 mg/mL; a molecule is defined “insoluble” at a maximum dissolved concentration ≤0.1 mg/mL [2,3]. Most of these drugs (70% of investigated molecules) are placed within the class II of the Biopharmaceutical Classification System (BCS), which includes molecules with poor solubility and high permeability through biological membranes. Molecules belonging to this category are further classified into the subclass IIa containing drugs that do not dissolve in water, but at least in some lipids or oils (i.e., fenofibrate), commonly called “grease ball” [4]. The second subclass IIb contains molecules also called “brick dust” that are insoluble not only in aqueous solvents, but also in lipids or oils due to their strong crystalline lattice energy. In order to improve their solubility, drugs included in the subclass IIb are commonly formulated as nanocrystals (NCs), since amorphization or the reduction in particle size through nanonization techniques could overcome or improve solubility issues [5]. The remaining 30% belongs to the class IV (BCS), which groups together molecules characterized by low permeability and solubility at the same time [6,7]. Drawbacks associated with poor solubility can lead to low drug bioavailability. Thus, different strategies have been explored to overcome insufficient drug solubility in water, including molecular modification carried out with salt formation, co-solvency or prodrug development; physical modification, including amorphous forms, co-crystalization, size reduction, micronization and nanonization, up to the design of drug delivery systems (nanoparticles (NPs), polymeric micelles, cyclodextrin inclusion, liposomes, microemulsions and self-emulsifying drug delivery systems) [8,9,10]. One of the most useful approaches to improve drug bioavailability consists of the reduction in particle size [11]. The variation from the micrometer to the nanometer scale through nanonization techniques leads to improved drug saturation solubility due to increased dissolution pressure of strongly curved small NCs according to the Ostwald’s ripening effect, which relates the curvature of particles to dissolution, whereby smaller particles (with a higher radius of curvature) are more soluble than larger ones [12], with improved dissolution rate due to increased surface area and enhanced adhesiveness of nanomaterial due to the increased contact area of small versus large particles [13,14,15,16]. Nanocrystalline drug technology allows to modify the physico-chemical properties of the drug with beneficial effects on its bioavailability. Furthermore, due to high drug loading, NCs maintain a potent therapeutic concentration to produce desirable pharmacological actions and can be very useful in the treatment of central nervous systems (CNS) diseases. In this review, the therapeutic applications of NCs by different administration routes is analyzed, among which the intranasal route is classified based on the local, systemic or brain delivery purpose. Special attention is dedicated to the brain delivery focusing on the therapeutic and diagnostic field. Recent studies on drug NCs aimed to treat the most common CNS pathologies (i.e., Parkinson’s disease, psychosis, Alzheimer’s disease, etc.) are discussed. Then, the recent use of fluorescent NCs in brain diagnostics, for the identification of cerebral vessels and tumor cells, is considered. Finally, the approved NCs for neurological disorders on the market are investigated.

## 2. Nanocrystals

NCs are defined as nanosized particles of pharmacologically active substances, with a crystalline (including amorphous form) characteristic [17]. NCs are essentially composed by a drug surrounded by a stabilizer, such as polymers or surfactants [18]. Among the polymeric stabilizers, poloxamers, such as Pluronic F68 and Pluronic F127, and polymers, such as polyvinyl alcohol (PVA), polyvinylpyrrolidone (PVP), hydroxypropyl methylcellulose (HPMC), and hydroxypropyl cellulose (HPC), have been the most widely used. Among surfactants, Tween^®^ 80 (non-ionic), sodium lauryl sulfate (SLS), sodium dodecyl sulfate (SDS), dioctyl sodium sulphosuccinate (DOSS) and others, such as Brij 78, lecithin, and TPGS 1000, have been used [19,20]. 

**Figure 1:**
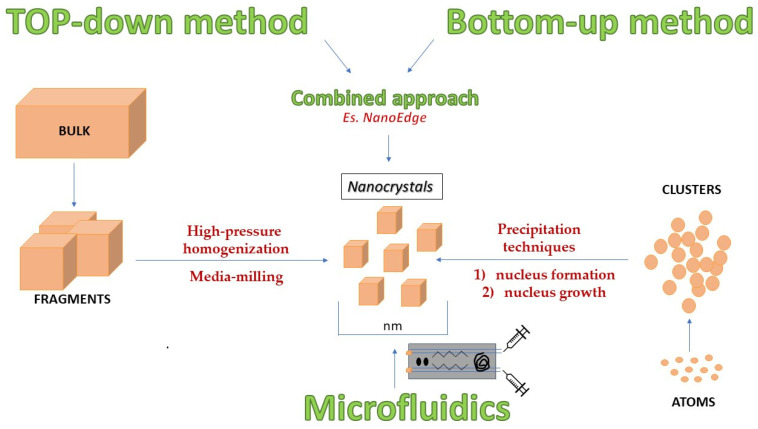
Main nanocrystal production techniques: Top-down method, Bottom-up method, combined approach, and the new microfluidic technique.

NCs are commonly referred as “nanosuspensions” since they can disperse in an aqueous phase [21]. NC production methods are classified into two major groups: top-down and bottom-up techniques (Figure 1). The top-down approaches involve the reduction in the bulk particle size down to the nanosize range using high energy approaches, such as high-pressure homogenization (HPH) and media milling (generally conducted in a liquid environment and leading to the formation of a nanosuspension) [22]. Bottom-up approaches are essentially precipitation techniques as the nanosized drug particles are obtained after precipitation from a supersaturated drug solution [23]. In addition, combination processes can be used in which both techniques (top-down and bottom-up) are considered, for example using one of the two methods as a pre-treatment (precipitation) and then moving to the second for size reduction (with the use of HPH or ultrasound). Among the approaches involving the combination of two methods, one patented technique is Nanoedge^®^, which couples a solvent/anti-solvent precipitation phase with an HPH annealing step [24]. This strategy is useful to avoid the limits of each single method (e.g., removal of the organic solvent in the bottom-up method) and to combine the advantages of each one (for instance, the reproducibility and industrial scale-up typical of top-down methods) [25,26]. Recently, a new method for NCs preparation has been proposed exploiting the microfluidic pathway. Specifically, it joins the fields of science and engineering, associated with a decrease in production costs with the simplicity and speed of the method. The use of micro-channels with a high area-to-volume ratio allows for the fabrication of micrometric and nanometric particles, bubbles, droplets, emulsions and liposomes [27]. 

Due to high drug loading, NCs maintain a potent therapeutic concentration to produce the desirable pharmacological actions, which can be exploited for several applications.

## 3. Nanocrystals: Routes of Administration

The possibility of adapting NCs to different pharmaceutical forms has led to the development of these nanosystems for different routes of administration, among which the most studied is the oral route. Examples of NCs design, development, and characterization for different delivery routes (oral, ocular, parenteral, pulmonary, dermal and intranasal) are reported in the following sections. The discussed routes of administration are the most common used for the delivery of NCs. 

### 3.1. Oral Route

The oral administration of drugs is the most used method of drug therapy because it is considered the safest and preferred route by patients. However, some challenges are associated with oral drug delivery, such as the hepatic first pass metabolism, the presence of enzymes and an unfavorable pH in the stomach that can inactivate the drug as well as inter-individual characteristics due to oral diseases, gastrointestinal damage, the phenomena of malabsorption, etc., that can affect the absorption and bioavailability of the API. Some of these issues could be overcome by means of the oral administration of the drugs as NCs. An example is given by the fact that NCs can overcome the local gastrointestinal (GI) damages caused by many anti-inflammatory drugs [28]. Compounds, such as meloxicam, can produce gastric lesions when administered orally due to the high concentration or repeated doses of the drug. The oral administration of meloxicam in the form of NCs could improve drug bioavailability, reducing or avoiding the repeated drug administration with the consequently reduction in drug toxicity in the GIT [29]. Xu et al. demonstrated the improved efficiency of cinacalcet NC capsules compared to conventional cinacalcet hydrochloride tablets, evaluating the dissolution rate in four media (pH 1.2 hydrochloric acid buffer solution, pH 4.5 acetic acid buffer solution, pH 6.8 PBS and water). The values of accumulated dissolution were all more than 95% without the effect of pH compared with the commercial tablet Sensipar^®^ and raw material [30]. One of the main advantages of NCs is that they can be formulated in a great variety of pharmaceutical forms, including easy-to-administer ones, such as NC-based oral film strips. The NC strips are also useful when it is necessary to formulate an API that must act quickly, releasing the drug either by chewing or in contact with salivary fluid in a short time [31]. This strategy could be very useful to treat patients affected by oral pathologies, with difficulties in swallowing or for pediatric patients. Studies showed that formulations based on NCs (tablets and capsules) exhibit a better drug-kinetic profile compared to the raw drug and conventional forms on the market. A pharmacokinetic study performed by Li et al. on nimodipine NCs demonstrated that this antiarrhythmic drug, when administered orally in the form of NCs, had a bioavailability that was 397% greater than that of conventional tablets (Nimotop^®^). Specifically, the conventional form of nimodipine dissolved quickly and underwent supersaturation, precipitating in the GI fluid and reducing the overall bioavailability of the drug. NCs, on the other hand, adhere to the wall of the GIT releasing the drug gradually and dissolving slowly, giving a more controlled release of the drug [32]. NCs of the beta-blocker carvedilol stabilized by alpha-tocopherol succinate (VES) showed increased dissolution rate and bioavailability compared to the commercial tablets [33]. The shape of NCs can also influence parameters, such as mucus permeation, transport through epithelial cells and bioavailability. NCs with rod-shape characteristics showed a significant cellular absorption and a greater epithelial transport than NCs with spherical shape, probably due to the larger surface area of elongated particles that increases the contact area between NCs and the cellular membrane [34]. Particle size is an influencing parameter in the bioavailability of orally administered drugs as reported by several studies; a twenty-fold increase in bioavailability between raw drug and NCs and a five-to-six-fold increase between micrometric and nanometric particles has been reported. An optimal size for increased oral absorption could be between 200 and 600 nm. The dissolution rate, the biodistribution and the oral bioavailability of coenzyme Q10 NCs was clearly affected by particle size. For NCs in a 120 to 700 nm size range, higher C_max_ and AUC_0–48_ values were registered in the smallest Q10 NCs [35]. In any case, the reduction in size below the micrometer range offers numerous advantages in terms of greater bioavailability [36]. 

In addition to particle shape and size, another factor that can influence the bioavailability of oral drug-loaded NCs is the stabilizer agent. It has been widely demonstrated that the type and concentration of stabilizer influence the size and stability of NCs, but it is also true that they can positively or negatively affect the in vivo drug performance. Mu et al. prepared spironolactone NCs with four different stabilizers, showing that the use of ionic stabilizers individually is not recommended. The use of a charged stabilizer alone increases the possibility of agglomeration between the NCs in the GIT, mainly due to the different pH variations. Sodium deoxycholate (NaDC) was used as stabilizer agent for spironolactone NCs: it is converted into insoluble deoxycholic acid at the acidic pH of the stomach, leading to the formation of aggregates that were not redispersed even in the intestinal pH 6.8. On the other hand, the in vivo performance was enhanced by spironolactone nanonization using stabilizers, such as Pluronic^®^ F127 and F68, and HPMC-C5. All of them gave similar results in terms of improved dissolution and bioavailability compared to the raw drug [37]. In other studies, the use of stabilizers, such as PVP, ethylcellulose or the combination of HPMC and PVA, produced an improvement in oral drug bioavailability [38]. Additionally, in the study of Tian et al., focused on the preparation of NCs with a multicomponent inartificial compound (Bufadienolides) with antitumour activity, it was demonstrated that different stabilisers caused multiple different mechanisms of NC endocytosis, intracellular transport, and transmembrane transport. The choice of an anticancer molecule as model drug is due to the fact that the bioavailability of orally used chemotherapeutic drugs is hampered by the difficulty of effectively penetrating through the mucus layer and transport layer of intestinal epithelial cells [39].

The combination of two types of innovative formulations (emulsion and NCs) into one is of great interest for improving the oral bioavailability of a drug. In the study by Zhang et al., NCs were incorporated into a self-stabilizing Pickering emulsion for oral administration. A Pickering emulsion is a special emulsion that uses ultrafine solid particles as emulsifier [40]. In this study, NCs were the solid particles that acted as emulsifier. The study showed that the relative bioavailability of a puerarin NC emulsion was significantly higher than of the crude puerarin suspension (262.43%), the NC suspension (155.92%) and the surfactant emulsion (223.65%), proving that the combination of these two types of formulation (NCs and Pickering emulsion) increased the oral bioavailability of the drug and, at the same time, the NCs are able to stabilize the emulsion [41]. Two recent studies focused on the preparation of NCs of irbesartan for oral administration. The first study by Deguchi et al. focused on the preparation by media milling of NCs coated with methylcellulose with a size less than 200 nm. To overcome stability issues, a second study by Nagai et al. was conducted to obtain a solid oral pharmaceutical form. Indeed, the NCs were then incorporated into tablets through drying approaches, resulting in a particle size of 118 nm after redispersion of the tablet [42,43]. NCs of naringenin, a molecule recognized for its anti-inflammatory activity, have been studied for the oral treatment of rheumatoid arthritis. Compared to the crude drug, the NCs showed dissolution behaviour, increased cellular uptake, and improved transcellular diffusion in comparison to the bulk drug naringenin. In vivo tests in rats demonstrated an improvement in rheumatoid arthritis in collagen-induced arthritic rats by reducing the infiltration of inflammatory cells and synovial damage [44].

### 3.2. Ocular Route

Ocular drug delivery is a major challenge due to the unique anatomy and physiology of the eye. The amount of drug that enters the anterior chamber is about 5% of the administered dose. There are numerous obstacles that have to be considered for this route, including the small volume that can be administered due to the conjunctival sac, which holds a volume of about 7 microliters, nasolacrimal drainage, eye barriers, blinking and lacrimal reflexes, irritation, and the tolerability of an instilled formulation. All these issues could be improved by exploiting NC technology [45]. Studies related to the ocular delivery of drug NCs are mainly directed to the treatment of inflammatory diseases and ~15% to glaucoma [46,47,48]. The most widely used ocular dosage form is the eye drops. As mentioned, one of the main limits is the small volume that the conjunctival sac can hold, which leads to the expulsion of most of the administered formulation. Researchers have tried to overcome this issue by producing formulations capable of increasing the residence time of the eye drops. The choice falls on increasing the mucoadhesive properties of the formulation, by using suitable stabilizers or by manufacturing positively charged NCs in order to increase their adhesiveness on the negatively charged ocular surface. This strategy was made possible by the use of stabilizers, such as cetylpyridinium chloride and benzalkonium chloride [49]. Another approach is to increase drug residence time by producing viscous solutions that adhere better on the corneal surface. The increase in viscosity, in fact, leads to a decrease in drug transport with the tear fluid and therefore to a lower drainage speed. One strategy is to prepare in situ gelling systems, which are normally administered as a solution that becomes a gel due to a change in electrolyte concentration, pH or temperature at the application site. In the study of Nagai et al., an in situ gelling system was prepared based on indomethacin NCs stabilized by Pluronic^®^ F127, characterized by a specific sol–gel transition temperature. This behavior could increase the residence time of the drug on the ocular surface increasing its permeability and bioavailability. The authors found that the concentration of the stabilizers played a crucial role since F127 at concentrations of 5% and 10% improved the drug permeability through the corneal epithelium, and this result was not achieved at higher (15%) stabilizer concentration [50]. Overall, it has been demonstrated that the use of NC technology can exert a double benefit to improve drug ocular administration, A short-term advantage is created due to the immediate increase in the drug mechanism of action, which is obtained by increasing the dissolution of the drug, with a consequent increase in the amount of drug available for absorption and the possible reduction in ocular toxicity. The long-term advantage consists of the prolonged release of the drug over time, given by the mucoadhesive NC properties that improve the NC ocular retention time and prolong drug action [51]. Therefore, the selection of suitable stabilizers and an optimal particle size is considered essential for ophthalmic NC administration since particles with size less than 200 nm are preferred to reach the retina district, and safe and well-tolerated stabilizers, such as PVP, PVA, HPMC, and poloxamers, are required to avoid ocular irritation [52,53]. Particle size exerts a significant impact on drug concentration in the tear fluid and on the ocular bioavailability of drugs from topical suspension. A nanosuspension of indomethacin with small particle sizes showed approximately a two times higher drug release into the aqueous humor than suspensions with larger particles (>1000 nm). Particle size affected the drug absorption of formulations with the same viscosity [54,55].

### 3.3. Parenteral Route

Two main issues are generally associated with drug administration via parenteral routes: toxicity and target site achievement. NCs have been studied for this purpose, and the choice of the stabilizer agent is crucial since not all stabilizers are suitable due to their toxicity. Ahire et al. showed a list of stabilizers allowed for parenteral administration: in addition to poloxamers, D-α-tocopheryl polyethylene glycol succinate (TPGS), amino acid derivatives, such as albumin and leucine, at concentrations ranging from 2% to 52.6%, respectively, or others, such as arginine and proline, can be found. NC mean size is another relevant point for parenteral administration, since particles should possess a diameter in the range of 100–300 nm [56]. As reported in the literature, it has been demonstrated that, after parenteral administration, curcumin NCs with a size <100 nm were poorly captured by cells, while particles with a size >500 nm were phagocytosed by macrophages. In fact, after injection, NCs can be recognized and captured by the mononuclear phagocyte system (MPS) and passively deposited in the liver, lung and spleen (organs rich in MPS) [57]. Changes in the NC surface can negatively or positively affect the attack by the endothelial reticulum system (RES): coating with serum albumin or dextran leads to increased recognition by the immune system and subsequent deposition in the liver and spleen. Instead, a PEG coating limits this event, justifying the fact that, in some cases, the fabrication of “stealth” NCs is relevant [58]. NC coating with cationic lipids, such as DOTAP or IGg, leads to greater recognition by the immune system and by monocytes of the CD14 and CD16 line. By exploiting this mechanism, it is possible to design NCs that can be transported by macrophages into cancer cells [59]. NCs, generally constituted by 100% of the drug, allow a high drug bioavailability from the first administration. Furthermore, the low concentration of stabilizers used avoids toxicity problems related to the parenteral administration of formulations containing higher quantities of surfactants [60]. The FDA approved Tween^®^ 80 (up to 10%) and Poloxamer^®^ F188 as stabilizers for injectable nanosuspensions, whereas TPGS is considered safe, but is more expensive [56]. 

Another strategy to improve drug targeting is to design pH-responsive NCs. This approach is useful to target the drug to an inflamed tissue or, for example, to a cancer cell in which the environment is acid. In this case, the NCs can be coated, for example, with calcium carbonate, which releases the drug in a pH-dependent manner. In this way, a low release at physiological pH and a rapid release at acid pH can be established, simulating the tumor environment [61]. NCs have been investigated also for peritoneal route, which consists of the administration of drugs directly into the peritoneal fluid. Intraperitoneal NCs are beneficial for the administration of chemotherapy drugs also known as PIPAC (pressurized intraperitoneal aerosol chemotherapy). It is carried out by administering a highly concentrated liquid NC formulation that is transformed in the form of aerosol and coats the abdominal cavity by increasing the diffusion coefficient and the final efficacy of the drug [62]. NCs were also studied for the hyperthermic intraperitoneal chemotherapy (HIPEC) strategy of paclitaxel [63]. In another study, a hyaluronic-acid-based hydrogel, containing paclitaxel NCs, was produced. The goal was to create a chemotherapy depot system, which would allow the slow and localized release of the molecule. In this way, the severe effects associated with conventional anticancer therapy could be avoided [64]. 

A recent study by Ancìc et al. focused on the design of resveratrol NCs for intraperitoneal administration, for a potential anticancer treatment of Ehrlich ascites tumor (EAT). In vivo studies in EAT-bearing mice showed that the administration, having a low systemic toxicity, led to a significant reduction in tumor cell proliferation in the abdominal cavity, and a reduction in angiogenesis in the peritoneum, demonstrating low risk and the high beneficial effects associated with resveratrol NCs [65].

### 3.4. Pulmonary Route

The studies on NCs for pulmonary drug delivery have grown over the past decade. The main advantages deriving from this pathway are the large pulmonary surface, the thin barriers, the low enzymatic activity, the high vascularization, and the possibility of avoiding the first pass hepatic metabolism. NCs can overcome many issues typical of conventional pulmonary formulations, including poor release profiles of hydrophobic drugs, different particle size and heterogeneity in the conventional aerosolized formulations and the intense drug clearance by the alveolar macrophages that eliminate particles larger than 1 µm. Thus, NC technology becomes advantageous, providing a minimal use of excipients in the formulation, the reduction in particle size with a major surface area exposed to lung lining fluids and the possibility to obtain prolonged drug release. The ability of NCs to settle in the lung and escape from mucus entrapment is mainly due to the particle size, which is sufficiently small to evade steric obstruction by the dense environment of mucin fibers, the shape (rod-like) and the type of stabilizer used that should be sufficiently muco-inert to evade association to mucins. In the work of Costabile et al., NCs coated with PEG improved particles diffusion in the mucus layer since the presence of a PEG moiety formed a brush layer on the NC shell, which reduces their adhesion to mucins [66,67]. As mentioned, for the pulmonary route, particle size plays a pivotal role, affecting the lung deposition of an aerosol and influencing the clinical effectiveness of a drug. A recent study demonstrated the correlation between the size and bioavailability of drug NCs. In particular, curcumin NCs with different sizes were analyzed. Small-sized NCs with dimensions up to 250 nm showed a great diffusion profile, through a model of mucus layer by Franz cells, compared to NCs with a medium (around 500 nm) or large size (1089 nm). The reason was attributed to the pores of the respiratory mucus layer with a diameter of ~200 nm, as particles with a diameter less than 200 nm penetrated the mucus layer, while the others were trapped in it. Similar findings were obtained with Calu-3 cell lines selected as bronchial epithelial cells [68]. Furthermore, it has been shown that crystals with a particle size below 300 nm are able to escape mucociliary clearance and alveolar macrophages and present a better lung retention compared to microparticles. For these reasons, the ideal particle size should be in the range below 300 nm to avoid elimination through mucociliary clearance and improve penetration through mucus [69]. Curcumin-based NCs have been designed to be delivered as dry inhalation powder (PPE). They are prepared by the wet milling method followed by spray drying, obtaining a useful formulation for the deposit of a good amount of drug in the lung, avoiding the problems of toxicity derived from the deposit of the drug in other sites. The final formulation was characterized by good flow properties due to small particle size and spherical shape, as shown in the SEM analysis, as well as good aerodynamic properties to ensure maximum bioavailability by inhalation, according to the fact that the ideal aerodynamic particle size of less than 5 μm improves the deposition in the lung [70]. NCs prepared with hyaluronic acid (HA) directed through the pulmonary pathway to lung cancer cells were obtained to target breast cancer cells through the CD44 receptor that has a binding domain for HA overexpressed in cancer cells [71]. Another study was focused on the design of NCs coated with mannose as a fluorescent probe with the aim of detecting the cancer cells that overexpressed the mannose receptors [72]. Other strategies are well summarized in the work of Kumar et al. [73].

### 3.5. Dermal Route

NCs administered via the skin are an advantageous strategy for water-insoluble molecules because they increase the degree of dissolution of the drug. This involves an increase in the concentration of the drug in solution on the side opposite to the targeting site and therefore an increase in its passage through the skin by a concentration gradient. This is advantageous to limit the application of the drug only to the affected area, thus reducing possible side effects. This is the case, for example, of the cortisone drug since the formulation through NCs improves its bioavailability and can be considered an alternative to the oral route. For example, in the work of Lohan et al., the authors compared a conventional cream formulation of dexamethasone with a dexamethasone NC-based formulation that promoted drug penetration through the stratum corneum, increasing drug bioavailability and efficiency [74]. The preparation of antioxidant-based nanosuspensions, incorporated into creams or gels, is a valid alternative in the field of cosmetics or aesthetic medicine. The topical administration of flavonoid-based NCs and antioxidant molecules, such as lutein, quercetin or hexperitine, preserves their biological properties, increasing their poor bioavailability [75,76,77]. Diosmin NCs were the constituents of wafers designed for the treatment of diabetic ulcers, combining the advantages of the adhesion of the pharmaceutical form (based on gelatin and alginate) with the increase in bioavailability given by the formulation of NCs [78]. Slow-release formulations can be formulated with NC-based patches and, as an innovation strategy, suspensions that can be administered follicularly can be used. The hair follicle has, in fact, proved to be an excellent reservoir for the containment and slow release of drugs, when dimensions that vary in the range between 400 and 700 nm. In particular, a study revealed that the increase in penetration through this path is due to the moisturizing characteristics that are provided on the skin by the excipient used for the preparation [79]. NC technology allows the preparation of dermal formulations with low viscosity and with a good release profile, making the active ingredient easily absorbable even at concentrations below 0.2% [80]. Thus, they became very useful in the case of anti-inflammatory drugs, such as highly gastrolesive diclofenac, increasing its topical bioavailability, as alternative to the oral administration. 

The synergy between the use of micro-needles and nanosystems has enormous potential, as reported by several studies. Indeed, micro-needles promote the penetration of systems by creating microchannels on the skin surface. For example, in a study performed by Pireddu et al., the administration of diclofenac NCs by micro-needles was investigated. This association was found to be synergistic and valid to increase the bioavailability of the drug at the target site without obtaining the side effects of gastric injury typical of a nonsteroidal anti-inflammatory drugs (NSAIDs) [81].

### 3.6. Intranasal Route

In recent years, the intranasal route has emerged as an attractive approach in the treatment of various diseases due to its potential for multiple actions. Indeed, a drug deposited on the nasal mucosa can exert a local effect and/or be absorbed into the bloodstream, performing a systemic action. Once the molecule dissolves into the nasal mucus layer, absorption is facilitated by a large, highly vascularized surface area with relatively low enzymatic activity. The nasal route is used for the topical administration of molecules for local treatment, typically with anti-allergens and nasal decongestants. In fact, the application of the drug to the site of action carries a lower risk of systemic side effects, such as the typical drowsiness associated with the oral administration of antihistamines [82,83]. 

In addition to the local and systemic action, intranasal administration has been widely investigated for the possibility to serve as a direct transport route to the brain (“nose-to-brain delivery”) since the nasal cavity is the only portion of the body in direct contact with both the central nervous system (CNS) and the external environment [84]. Examples of the application of intranasal administration for local and systemic delivery are discussed in the following subsections. Considering the emerging interest in the nose-to-brain delivery, this topic is detailed in a dedicated section (Section 4).

#### 3.6.1. Local Intranasal Delivery

Intranasal delivery has been widely investigated for local drug NC administration as highlighted by several studies available in the literature. The work of Alshweiat et al. investigated the effect of intranasal loratadine NCs. The nanosuspension of this anti-allergic drug showed a 1.84-fold increase in bioavailability compared to the crude drug and a 5.54-fold increase in bioavailability compared to the oral formulation. Loratadine NCs were prepared through the precipitation–ultrasonication method and modified with HA to improve mucoadhesion in the nasal cavity. The final formulation, thanks to the HA, showed a prolonged contact time with nasal mucosa and an enhanced dissolution in the artificial nasal fluid. This study showed that both the NC formulation and the choice of intranasal route proved to be crucial in terms of increasing the bioavailability of the drug compared to the pure form and the oral formulation of loratadine [85,86].

Saindane et al. designed a nanosuspension of carvedilol incorporated in situ into a gelling nasal spray formulation. The ability to gelify in situ occurred by gellan gum, which became an ion-sensitive carrier in contact with the nasal fluid. The study showed that the nanosuspension improved the bioavailability of the drug compared to the carvedilol administered orally: the absolute bioavailability (F_abs_) for the nasal and oral formulations, examined through an in vivo evaluation of the pharmacokinetic profile, were 69.38% and 25.96%, respectively. The method used to prepare the formulation was the precipitation–ultrasonication method. Characterization demonstrated good rheological properties, thanks to the presence of 0.5% *w/v* gellan gum, which can provide a desired viscosity (286 mPas) and quickly becomes a gel when it comes into contact with the nasal fluid, and it is involved in a prolonged release profile for up to 12 h in relation to its concentration (an increase in the concentration of gellan gum retards the release pattern) [87].

The work of Kurti et al. showed the preparation of a meloxicam NC powder by the co-grinder technique, which consists of obtaining a powder by mixing drug and excipients in appropriate ratios without the use of an organic solvent. Different combination of stabilizers and the drug were evaluated, but the most promising results in terms of structure and size for potential intranasal systemic drug delivery were obtained with PVP and PEG 6000 [88]. A recent study of Hassan et al. investigated the local use of ivermectin for the treatment of olfactory disorders, such as anosmia and hyposmia, in patients with mild COVID-19. In this clinical study, in which 114 patients were involved, the drug was administered as a mucoadhesive intranasal nanosuspension spray to the upper airways, where a large viral load was found in the early stages of infection. A nanosuspension of ivermectin prepared by the nanoprecipitation–ultrasonication method, using stabilizers, such as Poloxamer 407 and Poloxamer 188, was placed inside nasal spray containers. Prior to filling them, the nanosuspension was incorporated into a mixture of mucoadhesive polymers, such as HPMC K15M, Carbopol 974P, and sodium alginate, until a homogeneous and viscous formulation was obtained. The goal of reducing viral load in the upper respiratory tract by providing a uniform distribution of the drug across the nasal mucosa was achieved [89]. 

#### 3.6.2. Systemic Intranasal Delivery

The use of the intranasal route to reach the systemic circulation, on the other hand, is a widely used practice in the context of emergency situations, since it allows the administration of certain drugs quickly and safely, avoiding the first-pass metabolism typical of the oral route. Current studies are mainly concerned with the nasal application of systemically acting drugs, such as analgesics, cardiovascular and antiviral drugs, and biomacromolecular drugs, such as peptide drugs or vaccines. Thanks to this route, it is possible to avoid the first-pass metabolism and severe enzymatic degradation by the oral route and, at the same time, reach, thanks to the great vascularization of the nasal mucosa, high concentrations at plasma level [90].

Regarding the systemic route via nasal delivery, Su et al. reported the design of a nanosized fluticasone propionate nasal spray formulation to overcome the limited nasal permeability. The transmucosal nasal route was investigated. The formulation was prepared by using Tween^®^ 80 as stabilizer with the method of HPH and demonstrated properties comparable to the product on the market, good stability without growth particle size phenomena up to 30 days and better results in the delivered dose uniformity (DDU) test [91].

The study by Zhu et al. focused on the design of armodafinil NCs by the anti-solvent precipitation method to be targeted to the systemic route via intranasal administration. NCs were incorporated into a hydrogel of PVP K90 by giving them a bio-adhesive matrix with good viscosity (thousands mPas), capable of remaining in the nasal cavity for more than 4 h allowing rapid drug penetration through the nasal mucosa. Pharmacokinetic studies showed that, after the intranasal delivery of an armodafinil NC gel, plasma and brain exposure to the drug was twice as high as after the oral administration of an armodafinil solution. Most importantly, armodafinil NCs administered intranasally as a NC gel demonstrated, in a mice model, an improvement in the cognitive function and the control of sleep deprivation [92]. 

In another work, the precipitation–ultrasonication method was exploited to prepare zaleplon NCs for intranasal delivery in powder form. Different formulations were designed to obtain particles with a size of 200 nm by mixing polymers, such as Soluplus^®^ and poloxamer, with mannitol. NCs exhibited a higher C_max_ after intranasal administration in rabbits, corresponding to a higher extent of drug absorption for intranasal NCs since the nanosization had a positive impact on enhancing the dissolution rate of zaleplon. The significantly improved bioavailability of 314%, after intranasal administration in rabbits compared to the oral tablet, proved to be a successful alternative to the marketed oral formulation that showed poor absorption from the gastrointestinal tract [93]. 

## 4. Nanocrystals for Nose-to-Brain Delivery

**Figure 2:**
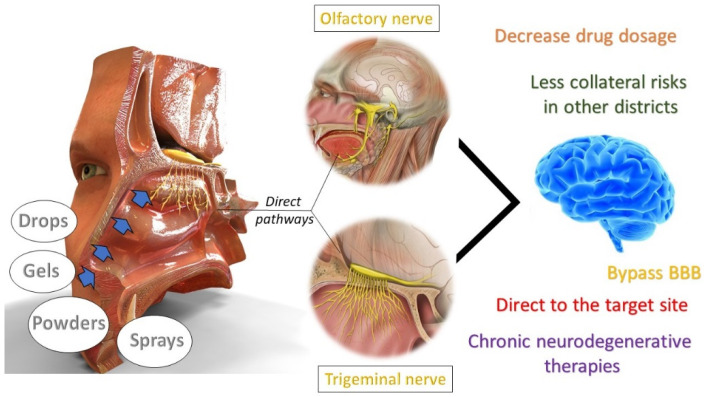
Strategies of direct drug delivery to the brain by intranasal administration: common pharmaceutical forms, pathways involved, and the challenges of nose-to-brain delivery.

The only direct access connecting the exterior environment and the CNS is the nasal cavity. The administration of drugs intranasally allows drugs to be conveyed to the brain overcoming the obstacle of the BBB. The intranasal route represents a non-invasive strategy for delivering drugs to the brain [94]. Different mechanisms seem to be involved in the transfer of molecules from the nasal cavity to the brain [95]. The two pathways that allow direct delivery through the nasal cavity to the brain are the olfactory nerve (the first cranial nerve) and the olfactory branch of the trigeminal nerve, as show in Figure 2. The olfactory pathway is characterized by the internalization of molecules into the olfactory sensory neurons by endocytosis or pinocytosis and their intracellular axonal transport along the neurons into the olfactory bulb, wherefrom the molecule is distributed throughout the CNS. Human olfactory axons have a typical diameter of 0.1–0.7 μm; thus, only molecules within such dimensions will be able to be transferred along this pathway [96]. The trigeminal pathway is less explored than the first one. The transport involves the three branches of the trigeminal nerves (ophthalmic, maxillary and mandibular) that innervate both the respiratory and olfactory mucosa, establishing a way for the delivery of drugs into the CNS [97]. In addition to the direct route, molecules with specific physico-chemical properties could also be transferred to the brain after intranasal administration through indirect transport. In fact, due to the high nasal mucosa, drugs can permeate and pass to the systemic circulation after nasal absorption [98]. The nose-to-brain route offers several advantages: quick and easy administration, reduction in side effects, and allows the administration of drugs overcoming the problem of first pass hepatic metabolism. Furthermore, the intranasal route is attractive and particularly useful for chronic therapies, especially for elderly or pediatric patients, as it may improve patient comfort, increasing compliance thanks to the easy self-administration [99]. The disadvantage is that it is influenced by interindividual characteristics (i.e., nasal congestion due to cold or allergic conditions, mucosal irritations, mucosal damage, irreversible damage of the cilia, complex geometry of the nose with a narrow passage, etc.) and the mucociliary clearance that restricts the volume of drugs that can be administered [84]. Accordingly, the administration of drugs to the brain via the intranasal route is limited to those drugs that are very potent at low concentrations, due to the small volume that can be given intranasally. Alternatively, as NCs are constituted by almost 100% drug, it could be considered a promising approach for this purpose. Several strategies have been exploited to improve nose–brain delivery, such as the use of nanotechnological strategies to enhance drug transfer to the brain. Drug encapsulation in phospholipid nanospheres has been exploited to increase drug bioavailability and passage through the BBB, or the use of positively charged polymer able to promote NP mucoadhesive properties, such as, for example, chitosan [100,101].

The most used pharmaceutical forms via the nasal route include powders, sprays, gels and drops [102]. In recent years, the nanotechnological formulations for the nose–brain route of administration that are proposed and patented have been steadily increasing. Only NC-based formulations developed for brain targeting through this route were considered. No patent was discussed because, to date, there is no evidence of patented formulations of NCs for nose-to-brain delivery [103]. The pathologies that these formulations have addressed are neurodegenerative and chronic diseases, such as Alzheimer’s disease (AD), schizophrenia, and Parkinson’s. Donepezil, a palliative molecule in AD, was formulated as a nanosuspension, with an average particle size between 100 and 200 nm. The study revealed that a well concentrated nanosuspension of donepezil (1.5 mg/mL) was effective in reaching the target site when administered intranasally, opening the door to further studies on this formulation [104]. Zotepine, an antipsychotic molecule, suffers remarkable hepatic first pass effect, which reduces the bioavailability of the drug. A zotepine nanosuspension was prepared, using a combination of two techniques: sonoprecipitation (SP) coupled with the HPH technique. Two different solvents and different stabilizers were tested to evaluate their influence on particle size [105]. As already mentioned, mucociliary clearance must be considered when administering drugs intranasally since this innate mechanism hinders the passage of the drug in the following areas, decreasing the dosage of the drug that finally reaches the target site. Therefore, innovative strategies are under investigation in order to increase the drug residence time in the nasal cavity to promote its absorption and diffusion. In order to improve the residence time of the formulation in the nasal cavity, the use of absorption promoters, the increase in the viscosity of the formulation or the design of in situ gelling systems have been proposed. Some evidence has shown promising results when NCs were incorporated into in situ gelling systems, where the gelling part was mostly composed of deacetylated gelling gum (DGG) considered as an ion-responsive agent [106,107]. In the study of Hao et al., a resveratrol nanosuspension was used for the preparation of a gelling system consisting of deacetylated gellan gum (ion-sensitive polymer), which gels in the presence of cations. This strategy allowed to increase the contact time of the drug with the nasal mucosa, avoiding easy drainage. It is important to achieve a balance in the final viscosity of the formulation because higher levels can impair the administration. The study yields an excellent result with an increase in brain targeting efficiency by over 400%, confirming the promising nose–brain pathway as a direct transport route [108]. Paeoniflorin, a molecule used in Chinese medicine, was studied in the treatment for Parkinson’s disease. Paeoniflorin NCs were prepared using TPGS as a stabilizer and their protective action on SH-SY5Y cells was evaluated. The results showed significant differences between the pure drug and paeoniflorin NCs since the NCs exerted a powerful neuroprotective capacity in reversing MPP + neurotoxicity (neurotoxin MPTP and its metabolite MPP + cause similar Parkinson’s symptoms), and increased paeoniflorin bioavailability compared to the pure drug [109]. The preparation of in situ gelling systems is greatly exploited for the ease of administration and the ability to significantly increase the drug residence time. Gellan gum was also used to prepare a formulation based on breviscapin as a model molecule. A breviscapin nanosuspension was prepared and incorporated into an in situ gelling system. Applied intranasally, it could act as an adjuvant in neurodegenerative diseases for its antioxidant effect [110]. One of the most recent studies was performed by Huang et al., in which harmine NCs were prepared by the combined technique of HPH and spray drying. The obtained nanosuspension was incorporated in a system based on DGG. The system was responsive to the Ca^2+^ ions present in the physiological liquids, therefore when administered in a liquid, it transformed into a viscous gel that remained in the nasal cavity, avoiding the easy drainage of the drug. NCs with a particle size of 100–200 nm were obtained, a range suitable for the delivery of drugs from the nose to the brain [111]. In the work of Bonaccorso et al., a formulation of curcumin NCs was designed thanks to the Design of Experiment (DoE) approach to explore the relationship between the independent variables (curcumin concentration, stabilizer concentration, solvent-to-antisolvent ratio and type of stabilizer) on the final NC mean diameter for potential nose-to-brain delivery. Curcumin was selected as a model molecule for its interesting pharmacological properties and belonged to the class IV of the BCS. NCs were optimized based on the desirability function and characterized from a physico-chemical, technological and biological point of view. The optimized formulation was finally tested on olfactory ensheathing cells (OECs), a particular type of glial cells that accompany the unmyelinated olfactory axon of receptor neurons. The comparative uptake study into the OECs between free curcumin and curcumin NCs revealed an efficient internalization of curcumin NCs into the cells, demonstrating that NCs can ameliorate drug permeability [112]. 

A collection of the discussed studies related to NCs application via different administration routes was reported in Table 1.

Considering that brain delivery is still nowadays an urgent challenge, special attention is dedicated to this topic in this review, with a general description of the main mechanisms of transport involved in drug delivery to the brain and a deep investigation of the current NC therapeutic and diagnostic applications available for brain disorders. 

## 5. Brain Delivery: General Considerations and Mechanisms of Transport

The aim of brain drug targeting is the delivery of therapeutics crossing or bypassing the barriers that protect the brain from the entry of foreign substances. The blood–brain barrier (BBB) provides both anatomical and physiological protection for the CNS. The BBB is a highly selective barrier consisting of three interfaces: the endothelial cells of the capillaries, the cerebrospinal fluid barrier consisting of the cerebrospinal fluid (CSF) and the interstitial fluid (ISF), and finally the arachnoid epithelium. In addition, the passage through the endothelial cells is hampered by tight junctions, which allow the passage of oxygen and small lipophilic molecules, but avoid the passage of hydrophilic molecules [113]. This barrier is a dynamic interface with a range of interrelated functions, resulting from extremely effective tight junctions, trans-endothelial transport systems, enzymes, and the regulation of leukocyte permeation, which thereby generates the physical, transport, enzymatic, and immune regulatory functions of the BBB [114]. The arrival of drugs to the brain is also influenced by the presence of the ATP-binding transmembrane ABC protein, which is involved in the transport of many substances [115,116]. There is no effective treatment for almost all the brain diseases. If, on the one hand, the BBB gives protection to the brain, on the other hand, it has a very selective permeability for bioactive compounds, which is one of the major challenges for brain drug delivery. Generally, only lipophilic molecules with a low molecular weight (MW) (under 400–600 Da) find no difficulty in passing the BBB. BBB permeation decreases 100-fold when the MW of the drug increases from 300 to 450 Da. The basic mechanisms by which solute molecules move across membranes are detailed in the study of Bellettato and Scarpa [117]. 

Therefore, the development of new therapeutic approaches for several neurological and neurodegenerative diseases is still nowadays a severe challenge. The difficulty to selectively direct the drug towards the target site, avoiding serious side effects due to the passage of the drug to other districts, is only one of the main issues. However, the biggest challenge is overcome, the barriers related to the CSN, which do not allow the passage of 95% of candidate drugs [115]. 

**Figure 3:**
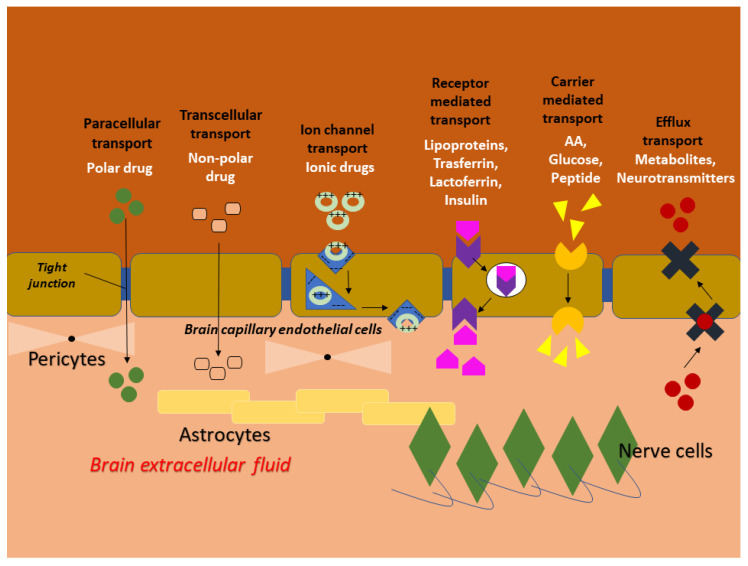
Schematic representation of drug transport across the BBB via different mechanisms, including paracellular and transcellular transport, receptor-mediated transport, ion channel transport of surface charged molecules, carrier-mediated transport of amino acids, glucose, larger proteins, and peptides, and the efflux transport that regulates the outflow of metabolites, drugs, toxins and neurotransmitters (Adapted from [118], NCBI, 2019).

In Figure 3, a schematic representation of the different pathways that are involved in the molecular transport into the CNS can be observed. For example, the transcellular pathway allows the passage of large molecules, such as sugars, amino acids, and fatty acids, but using transporters mainly present in the BBB, such as glucose transporters GLUT-1, LAT-1 and MCT-1, essential amino acids, and brain lactate, respectively. Large molecules, such as lipoproteins, lactoferrin or insulin, pass through the CNS through complex and poorly understood transport systems. Transport is mainly associated with receptor-mediated transcytosis (RMT). This transport involves the internalization of molecules into vesicles and entry through them [118,119,120].

**Figure 4:**
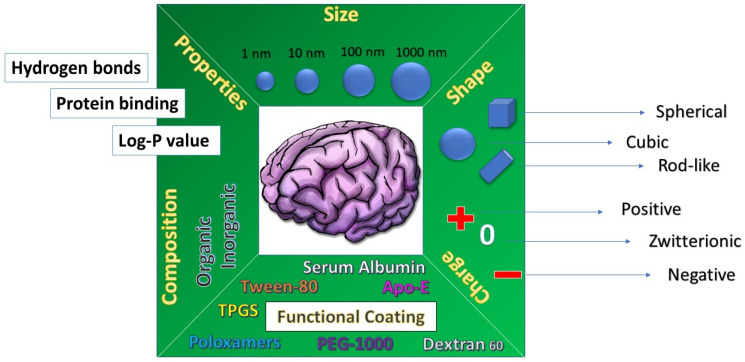
Properties that affect nanomedicine brain entrance and diffusion (Adapted from [125], Elsevier, 2016).

Drug delivery by nanotechnological formulations could promote brain targeting by different transport mechanisms, including the paracellular pathway, the transcellular pathway, the carrier-mediated pathway, receptor-mediated transcytosis and adsorptive transcytosis, cited above [121]. Furthermore, the transport of nanopharmaceutics is influenced by several physico-chemical properties, such as particle size, lipophilicity/hydrophilicity, surface charge, hydrogen bonds, degree of protein binding, shape, and functional coating, as represented in Figure 4 [122,123,124,125]. These properties affect the mechanism by which the molecules or the nanosystem can be internalized by the cellular compartment and can diffuse inside the brain. 

Among the nanosystems studied for brain delivery, NCs have shown great benefits, especially for their ability to provide a potent drug therapeutic concentration useful for achieving therapeutic action in brain diseases. With this in mind, the following sections consider studies in which the advantages offered by NCs over conventional treatments or other delivery systems are discussed and classified on the basis of the neurological disease. 

### 5.1. Nanocrystals for Neurological Diseases

Among the drug delivery systems direct to the brain, the most used are liposomes [126], microemulsions [127], NCs, lipomers [128], dendrimers [129], polymeric NPs [130], niosomes [131], solid lipid nanoparticles (SLN) [132], nanostructured lipid carriers (NLC) [133] and cellular vesicles [134]. Within these brain drug delivery strategies, we focused our attention on NCs. Most studies are focused on the treatment of Parkinson’s and schizophrenia, followed by Alzheimer’s disease, cerebrovascular diseases and brain damage, which are secondary consequences deriving from pathologies that affect other districts. Some of these neurological conditions were reviewed by Phuna et al. [135], thus additional literature studies were, in this review, reported in order to implement and integrate this recent review.

#### 5.1.1. Parkinson’s Disease

Parkinson’s disease is a neurodegenerative disorder that involves motor and balance functions in a slow but progressive way. It is a long-term disabling disease that mostly involves people over 50 years of age, involving the degeneration of dopaminergic neurons in the substantia nigra [136]. It has been demonstrated that the damage induced by free radicals is one of the causes that lead to the onset of a neurodegenerative disease. Accordingly, different studies investigate the role of antioxidant molecules for Parkinson’s disease treatment [137]. Therefore, the use of polyphenols and antioxidant molecules could improve the efficacy of the therapeutic treatment against Parkinson’s disease, even if their therapeutic application is often limited due to their poor bioavailability. Schisanterin, a Chinese medicinal herb, has been evaluated for its antioxidant properties and for reducing dopaminergic loss. Studies have shown that this molecule is able to cross the BBB, but its oral bioavailability is very low. In the work of Chen et al., schisanterin NCs were developed and showed a significant improvement of drug bioavailability compared to the pure drug. Neuroprotective bioassay conducted on zebrafish revealed that schisanterin NCs reverse MPTP (1-methyl-4-phenyl-1,2,3,6-tetrahydropyridine), a neurotoxic substance that produces the degenerative effects of Parkinson’s disease [138]. NCs made of ginkolide B, which is a molecule extracted from Gingko Biloba and characterized by a powerful antiparkinsonian effect, were prepared by Liu et al. The authors found that, thanks to their small size (less than 100 nm), the dissolution rate of the drug and the cellular absorption by endocytosis was significantly increased compared to the free drug. Furthermore, ginkolide B NCs showed greater bioavailability in vivo than coarse ginkolide B, leading to a potential reduction in drug dose for Parkinson’s patients [139]. In the study of Xiong et al., the authors focused on the improvement of the oral bioavailability of a molecule with strong antioxidant power, resveratrol. Resveratrol could be used as an adjuvant in the treatment of Parkinson’s diseases, which include conditions of oxidative stress. However, its huge benefits are hindered by its very low water solubility. For this purpose, resveratrol NCs were prepared with the solvent–antisolvent technique revealing an increase in drug bioavailability compared to the pure drug [140]. Very interesting results were obtained by Ghaffari et al., which investigated the effect of quercetin NCs on an induced model of oxidative stress. In this study, a parkinsonian model was induced in rats by using a toxic molecule, hydroxydopamine, capable of generating free radical stress. The administration of quercetin NCs, prepared with the evaporative precipitation of the nanosuspension technique coupled with HPH, reduced the levels of oxidative stress in the hippocampal area, protecting this area of the brain from degeneration induced by free radicals. This study, compared to the previous one, also confirmed the improvement of drug uptake. The antioxidant activity of NCs was confirmed by different assays, such as the determination of the quercetin NC effect on superoxide dismutase (SOD) and catalase activity (CAT), and the effect of neat quercetin and quercetin NCs on the total glutathione content and malondialdehyde level, revealing that the formulation restored the levels of SOD and CAT and increased the levels of GSH with a final decrease in lipid peroxidation, offering a wide contribution of antioxidant activity [141]. Xiong et al. formulated puerarin NCs, a medical herb that has therapeutic properties for various diseases, such as diabetes, Parkinson’s disease, and ischemic stroke. This molecule is placed within the class IV of the BCS system. Several strategies have already been attempted to improve puerarin solubility, such as puerarin encapsulation into SLN [142] or polymeric NPs [143], but none have achieved a satisfactory encapsulation efficiency, such as that obtained with NCs. In the work of Xiong et al., five different stabilizers were investigated for NC production and Pluronic F-68 was selected, as it maintained stability over time and in the fluids of the GI system and offered a higher drug dissolution rate [144]. In the recent work of Tan et al., a magnolol NC-based formulation (MAG-NCs) complexed with a hydrogel of poly(N-isopropylacrylamide) (PNIPAM) was designed for the treatment of Parkinson’s disease by intranasal injection. The formulation prolonged the residence time of the drug into the nasal cavity and decreased its clearance thanks to the processes of self-gelation at near-body temperature. Moreover, the nanometric size improved its crossing through the BBB by enhancing its delivery to the brain. In vivo tests in a mouse model of Parkinson’s disease with an intranasal injection of MAG-NCs demonstrated a reduction in neurotoxicity through the downregulation of ROS and ATP in dopaminergic neurons, typical of Parkinson’s mitochondrial dysfunction. Taking into account these findings, NC technology can be considered a useful approach to improve the antioxidant molecule bioavailability intended for Parkinson’s treatment [145]. 

#### 5.1.2. Psychosis

The main feature of psychosis lies in an altered perception and interpretation of the environment, false beliefs, and disorganized patterns of speech and behavior. In clinical practice, it is seen as a severe mental illness, in which delusions and hallucinations are predominant. Research has shown that elderly patients are at a greater risk for the development of psychotic symptoms. Therefore, good patient compliance is of outmost importance, considering that the therapy is mainly addressed to geriatric patients [146]. For example, long-acting antipsychotic formulations have been recently discovered, in order to increase patient compliance, reducing the number of administrations over time. Some of these have been approved by the FDA and consist in NC-based drugs for intramuscular administration [147]. Different efforts have been made in order to improve patients’ adherence to therapy. For example, adhesive buccal films are the most recently developed dosage form that can be easily administered and promote drug release in a controlled manner. An aripiprazole NC formulation was used to impregnate a double-sided adhesive film consisting of chitosan. Aripiprazole NCs were prepared by acid–base neutralization process and then incorporated into the polymer blend (chitosan and glycerol) before being cast and forming the film. Aripiprazole is a new generation antipsychotic drug, useful for chronic and long-term therapy. The film is simple to use and can be useful for the immediate delivery of the drug into the systemic circulation, thanks to the great vascularization of the buccal mucosa [148]. The work of Gol et al. involved the preparation of risperidone NCs (a second generation of antipsychotic drug), due to its high log P and its low solubility (class II BCS system). In particular, the authors investigated the impact of the process variables, including the solvent used, the drug-to-surfactant ratio, the solvent-to-antisolvent ratio and the stirring speed on the particle size and polydispersity index of the final risperidone nanosuspension [149]. Within the second generation of antipsychotics, NCs were also considered for ziprasidone, which is characterized by a poor oral bioavailability. The study focused on the preparation of ziprasidone NCs to obtain orodispersible tablets through direct compression. The technology of oral dispersible tablets (ODTs) is useful in the case of neurodegenerative diseases and in the treatment of elderly patients and children, overcoming swallowing difficulties. In this work, ODTs were prepared by microfluidization. The whole study was conducted using the Design of Experiments (DoE) approach to optimize the final formulation, investigating the ratios between disintegrants and lubricants, in order to gain a final formulation with optimal parameters of hardness, friability and disintegration time [150]. Another work on risperidone was carried out by preparing two different nanosuspensions using Pluronic F127 or PVP K30 by nanoprecipitation. The study demonstrated that the risperidone-based nanosuspension improved drug bioavailability. The Cmax and AUC curves, obtained from the in vivo investigation on rabbits, revealed a clear difference between the commercial form Risperidal^®^ and the risperidone nanosuspension, with a bioavailability increased by two times compared to the marketed product [151].

#### 5.1.3. Alzheimer’s Disease and Cerebral Ischemia 

Alzheimer’s disease (AD) is the most common form of dementia, with progressive and irreversible loss of cognitive function, recognized by the World Health Organization as a global public health priority [152]. Currently, the drugs are available for palliative care to improve patients’ quality of life. In addition, the low bioavailability of the drugs encourages and motivates the search for new strategies to increase treatment efficacy and to enable brain targeting to achieve the desired responses. As stated previously, the main limitation for the therapeutic treatment of neurodegenerative disorders, AD included, is the difficulty to reach the target site. The application of nanomedicine that delivers the drug directly to the brain could overcome this issue. However, this field has been little explored, since projects concerning the use of nanotechnologies to avoid the passage of the BBB for AD are less than 1% [153]. NCs with calpain inhibitors have been designed. Calpain has been shown to play a role in intractable diseases, such as AD. In AD, beta amyloid plaques lead to an increase in intracellular calcium, mediated by NMDA glutamate receptors, with a consequent final increase in this molecule. In neurodegenerative diseases as well as in AD, an increase in the concentration of calcium ions leads to the final activation of calpain, responsible for the apoptotic processes of cell death. Hence, calpain inhibitors can be useful in the treatment of AD [154]. NCs based on calpain inhibitors and more precisely on calpain I and SNJ-1945 inhibitors were designed for the first time by the spray dryer method [155]. One of the most used molecules in the treatment of AD is donepezil. Its use in the form of oral tablets causes a gastro-injurious effect that can be avoided after intramuscular administration. The intramuscular administration of donepezil improves its bioavailability and reduced peripheral side effects, but at the same time, it is associated with poor patient compliance, especially for long-term therapy, which can be improved thanks to the new long-acting formulations. At the same time, the donepezil orodispersible tablets called Aricept^®^ show the problem of severe gastrointestinal side effects and require daily dosing, compromising the adherence to the therapy. To overcome these problems, in the work of Mittapelly et al., donepezil NCs were designed for intramuscular administration, aimed at obtaining a slow release over time, in the manner of a long-acting formulation. NCs were prepared by the HPH technique, leading to a higher loading efficiency than the conventional formulation Aricept^®^ (less than 17%). In the study, donepezil NCs were prepared using an intermediate hydrophobic derivative by the hydrophobic ion pair (HIP) technique, prior to homogenization. The native donepezil is the second drug approved by the U.S. FDA for the treatment of mild-to-moderate AD, but it is a hydrochloride salt that has high aqueous solubility, so it is necessary to increase its hydrophobicity prior to NC formation. Embonic acid was chosen as adjuvant molecule with the aim of improving the hydrophobicity of donepezil through insoluble salt formation with the method of hydrophobic ion pairing. The study resulted in a stable formulation with a high drug load, which revealed good efficacy to replace conventional donepezil-based therapies [156]. The up-regulation of the group A phospholipase A2 enzyme (sPLA2-IIA) was found in rats after cerebral ischemia. This can be considered a marker in inflammatory diseases, such as atherosclerosis or sepsis, and neurodegenerative diseases, such as AD and cerebral ischemic stroke. Wang et al. focused on the potential treatment of cerebral ischemia with PX-18 NCs, considering the high production of sPLA2-IIA in the AD; PX-18 NCs could also be useful in the treatment of AD. PX-18 is considered a phospholipase A2 inhibitor. PX-18 NCs were prepared by HPH to obtain a “ready-to-use” formulation for parenteral administration. The formulation exhibited excellent stability, keeping the particle size unchanged for 180 days, which is promising for neuronal damage reduction [157].

#### 5.1.4. Brain Infections

A brain infection refers to an infection caused by viruses, bacteria, fungi, or parasites that affects the brain. Encephalitis is one of the most common examples referring to brain inflammation caused by bacteria, amoebas, viruses (such as cytomegalovirus) or parasites, such as Toxoplasma Gondii [158]. It has been reported that NC functional coating can improve NC transport, absorption and accumulation in brain areas as demonstrated on in vivo studies in rats via intravenous administration. Studies showed increased brain absorption using coating agents, such as polysorbates, poloxamers and/or peptides. It has been demonstrated, for example, that the modification of NC surface with polysorbate 80 allowed to bind the lipoproteins that act as receptor ligands for LDL receptors at the BBB level, inducing a better formulation absorption in the brain [159,160,161]. In the study of Dibaei et al., the authors demonstrated an improvement of curcumin biodistribution in the brain thanks to the design of coated nanosuspension due to the presence of Tween^®^ 80 or TPGS as a coating agent. The results revealed that the best coating agent was found to be polysorbate 80, which allowed drug absorption and targeting to the brain, confirmed by the plasma absorption of the protein ApoE on Tween^®^ 80. Curcumin NCs were recognized as LDL and interacted with endothelial cells in the capillaries of the brain and were absorbed via endocytosis into the brain. The literature suggested that peptides could serve as coating agents in order to improve drug targeting to the brain [161,162]. Accordingly, this strategy was investigated in some studies focused on atovaquone, used for brain toxoplasmosis. Toxoplasmosis is a zoonosis caused by the *Toxoplasma gondii* parasite and this pathology also affects the CNS. Two different studies were performed on atovaquone by using different coating systems to improve its brain targeting. The first study involved patients with AIDS, which were affected by manifestations of abscesses in the CNS (brain and cerebellum) and neurotoxicosis (Toxoplasma brain infection) with acute symptoms, such as seizures, paresis, or comatose states. Atovaquone NCs were formulated with three different surfactants Tween^®^ 80, poloxamer 184 or poloxamer 338 as absorption enhancers and the same NCs formulated with ApoE as coating agent, in addition to the three surfactants, and their brain targeting efficacy was investigated. The results showed that the ApoE-coated atovaquone NCs did not achieve the desired absorption in the brain, while the Tween^®^ 80-coated atovaquone NCs enhanced drug absorption to the brain compartment. The authors stated that the presence of ApoE improved atovaquone NC accumulation in the brain endothelial cells, but the enhancement of drug absorption in the brain was due to the presence of Tween^®^ 80 and the mean size of the NCs [163]. In a following study, the same authors prepared atovaquone NCs by using sodium dodecyl sulfate (SDS) or Poloxamer 188 for the treatment of toxoplasma encephalitis compared with the commercial drug Wellvone^®^. The first difference between atovaquone NCs and Wellvone^®^ (atovaquone micronized suspension on the market) was the particle size and polydispersity index, which was much higher for the marketed product. Significant differences were found related to the biodistribution of the drug. In particular, on day 12 of infection, mice were treated and drug distribution and absorption in organs, such as brain, lungs and liver, were assessed by HPLC analysis. Poloxamer-coated atovaquone NCs gave similar results to the commercially available Wellvone^®^ formulation, while SDS-Atovaquone NCs resulted in a very high concentration of the drug in the various districts considered, including the brain, compared to the commercial pharmaceutical form [164]. In the study of Lemke et al., amphotericin B NCs were investigated against encephalitis caused by the amoeba *Balamuthia mandrillaris*. Amphotericin B is characterized by low water solubility and nephrotoxicity. Seven nanosuspensions were prepared differing in the surfactant’s concentration. The most promising formulation was found to be that prepared with Tween^®^ 80 and sodium cholate (SC), promoting the high absorption of ApoE at the surface of NCs and a low absorption of opsonins, such as fibrinogen and IgG γ. The good absorption of ApoE on the NC surface and a minimization of hepatic absorption (low fibrinogen and IgG γ) indicated a high absorption in the brain [165]. A coating model was designed for Baicalin NCs, with the aim of obtaining a formulation for both ischemic stroke and Alzheimer’s disease. To improve the passage of the BBB due to the low bioavailability of the molecule, the nanosuspension, addressed to the intravenous route, was prepared using a combination of Tween^®^ 80 and TPGS as stabilizer agents. The final goal was to obtain a formulation able to absorbs ApoE on the surface, acting as a ligand for the LDL receptors present on the BBB, improving their crossing and leading to their brain-uptake by endocytic processes. As mentioned above, the coating with ApoE led to a lower adsorption of fibrinogen and Ig γ (opsonins) encountered in the blood that can promote phagocytosis and the removal of the drug carriers from the systemic circulation [166]. Serum albumin and polyethylene glycol 1000 were also investigated as functional coatings for neviparine NCs. Nevirapine is a non-nucleotide reverse transcriptase inhibitor used in the treatment of HIV-associated dementia. The problem with antiretroviral drugs is the difficulty of reaching the “reservoir” site or reaching it at low doses or for short periods of time. To be effective, they need high dosages, which inevitably leads to side effects. Nevirapine NCs were obtained by HPH. Dextran 60, PEG 1000 or serum albumin were absorbed on the NC surface as a functional coating to improve drug brain targeting. The best cellular absorption was obtained with serum albumin, 3.84 times greater than the pure drug and 1.39-fold greater than the uncoated NCs probably due to an increased recognition by macrophage receptors [58]. Therefore, the use of a coating agents can be defined “functional” when used to increase cellular absorption and drug concentration at the target site, avoiding the massive dosage of antiretroviral drugs.

#### 5.1.5. Brain Tumors

The effective delivery of chemotherapy drugs to the brain is very difficult, and the treatment must necessarily be targeted to the specific tumor site in order to avoid the drug’s accumulation in healthy tissues. The presence of coating agents on the particle surface could be useful to promote drug delivery to a specific district. Chai et al. designed a coated docetaxel NCs, modified with tumor-targeting ligands, for the treatment of glioma. Docetaxel is an antimitotic drug, and its use is associated with severe side effects, including aplastic anemia, mouth ulcers, nausea, vomiting and hair loss. The administration of docetaxel NCs could reduce drug dosage and the peripheral side effects. After preparation, docetaxel NCs were coated with a membrane constituted by red blood cells (RBCs) modified via avidin–biotin interactions by adding a tumor-targeting ligand (Rgdyk) that binds to integrins on cancer cells. The final formulation improved drug efficiency and targeting to the brain after intravenous administration [167]. In another study, 20(S)-protopanaxadiol NCs were prepared by the solvent–antisolvent precipitation technique. The 20(S)-protopanaxadiol is a molecule that has recently been re-evaluated for its potent anti-cancer action, comparable to paclitaxel. The 20(S)-protopanaxadiol NCs were then coated with TPGS. It has been shown that TPGS, safe and approved by the FDA, can induce apoptosis and implement the effect of some chemotherapy molecules [168,169].

#### 5.1.6. Demyelinating Pathologies

Nerves are mostly covered by a myelin sheath, which consists of lipids and proteins and can be considered a protective layer that wraps around the axons of neurons to aid in the insulation of the neurons, to increase the number of electrical signals being transferred and enhance the speed of these electrical signals, allowing all actions to be conducted quickly. The process by which myelin is formed is carried out by Schwann cells and oligodendrocytes. The process requires a lot of energy and metabolic precursors, such as acetyl-coenzyme A, pyruvate, and NADPH. Thus, in conditions of metabolic stress, the myelin formation process fails and the reduced ability to form it leads to pathological conditions [170]. Multiple sclerosis (MS) is one example, in which the myelin coating around axons and the ability to transmit neuronal signals is lost, followed by the death of neurons. Studies show that markers of metabolic stress have been found in MS. In fact, in conditions of metabolic stress, oligodendrocytes tend to reduce the production of myelin to safeguard cell survival [171]. Some remyelination strategies are discussed In the following. The administration of gold exerts beneficial effects on cellular metabolism. The study of Robinson focused on the preparation of a nanosuspension based on gold NCs with a clean and faceted surface, revealing that these systems, having a mean size of about 80 nm, can be powerfully catalytic. They catalyze an important reaction of the cellular metabolic process, namely the oxidation of NADH, becoming important for the generation of ATP, glycolysis and oxidative phosphorylation. Thus, gold NCs can be useful in cases of remyelination or diseases, such as MS, to reactivate the metabolism useful for the production of myelin. Additionally, the comparison between bulk gold and gold reduced to nanometric dimensions (NCs) revealed that, only in this latter condition, it exerted a catalytic capacity [172]. Curcumin-based NCs were prepared for administration to rat models of the MS disease. In the study of Caillaud et al., curcumin NCs were administered intraperitoneally to an animal model of the transgenic Charcot–Marie–Tooth disease (CMT type 1A). Curcumin was selected for its antioxidant power. Neurodegenerative diseases, such as CMT, are often associated with the aberrant folding of a protein derived from oxidative stress; therefore, it is advantageous to use a molecule with a powerful antioxidant effect. A previous study showed that the administration of curcumin near the damaged nerve allowed for slight repair. In addition, this molecule can stimulate the remyelination of demyelinated nerves. Curcumin NCs can be used in the treatment of the CMT disease, a disease associated with damaged nerves and damaged nerve conduction, as well as with muscle weakness and atrophy. Cellulose NCs functionalized with β-cyclodextrin were prepared. The results of the study showed that the formulation of curcumin NCs led to a decrease in reactive oxygen species (ROS), an increase in nervous performance, a decrease in creatinine (marker of muscle lysis) and an increase in antioxidant enzymes. Thus, the authors considered the formulation promising for the treatment of demyelinating pathologies [173]. Studies based on NC technology discussed in the previous subsections were grouped based on the pathology of interest in Table 2.

### 5.2. Nanocrystals in Brain Diagnostics

NCs made with fluorescent materials can be used in diagnostics and exhibited potential for detecting damaged vessel or cancer cells. Due to their higher brightness, higher photostability, and narrower spectral emission than conventional organic fluorophores, fluorescent semiconductor NCs are increasingly used in high-resolution cell imaging, as well as for tumor targeting and diagnostics. The main difference between fluorescent NCs and drug NCs is their chemical composition, since fluorescent NCs are composed of inorganic materials (gold, cadmium telluride, cadmium selenide, and iron oxide), while drug NCs are made of organic stabilizers (i.e., poloxamers, PVP, etc.) and APIs. Different methods of preparation can be used to obtain fluorescent or drug NCs that differ also in their physico-chemical properties (charge, spectral profile, colloidal stability, and magnetism) [174]. For example, fluorescent NCs are characterized by a particle size ranging from 2 to 100 nm, while drug NCs generally range from 1 nm to 1000 nm. Colloidal fluorescent semiconductor NCs, also known as quantum dots, present unique optical, electronic and photophysical properties that make them appealing in biological labeling, imaging and detection [175]. The following studies are based on brain diagnostics using NCs for brain lesion imaging or studies related to diagnostics for CNS-related diseases.

#### 5.2.1. Rare Earth Nanocrystals

Many studies concern NCs made of elements of the lanthanide series, commonly known as rare-earth elements. Among these, erbium, ytterbium and europium are the most used in the preparation of NCs for brain diagnostics. The work of Fan et al. summarizes the current landscape regarding bioimaging using luminescent probes consisting of NCs based on lanthanides, which is a non-invasive diagnostic with emissions in the NIR II region (second region of the near infrared). In particular, the authors referred to the use of nanoprobes for the diagnosis of cerebral vascularization [176]. One of the main disadvantages of most diagnostic NCs is the high toxicity due to the presence of heavy metal In the study of Choi et al., “heavy-metal-free” fluorescent NCs for intranasal administration were prepared. As detailed in Section 4, this administration route is gaining great attention for its feasibility to serve as a direct transport to the brain, bypassing the BBB and allowing the possibility to detect ischemic brain lesions through fluorescent NCs. This strategy could overcome the degradation and agglomeration associated with the intravenous and/or subcutaneous administration of NCs due to the superficial absorption of blood proteins. An advantage is that NIR NCs fluoresce in the near infrared region, so that they do not absorb skin or other organ-derived fluorescence. In order to improve their water solubility and transport, NIR NCs were coated with PEG in the final step, allowing for brain injury detection and damage caused by neurodegenerative diseases. Finally, these cadmium-free NCs allowed for repeated and long-term diagnoses [177]. The imaging of brain vessels through luminescence-emitting NCs is one of the main studies conducted in this field. In the work of Zhong et al., rare-earth NCs were prepared and then doped with cerium emitting luminescence at 1500 nm. In contrast to near infrared (NIR I, 500–900 nm), their use enhances tissue resolution and the quality of in vivo imaging diagnostics. They have shown promising results for their non-toxicity and photostability in aqueous fluids, which makes them stable in biological fluids. The test was performed on buffered saline solutions and bovine serum. NCs with dimensions of approximately 18 nm were synthesized by the co-thermolysis of rare-earth trifluoroacetates into oleic acid, 1-octadecene and/or oleylamine. They were doped with Ce^3+^ to reach a luminescence of 1500 nm. To make the surface hydrophilic and biocompatible, poly-(maleic anhydride-alt-1-octadecene, PMH) was used, which was bonded through van der Waals bonds using a catalyst to the oleic acid present on the surface. The formulation, injected intravenously, excited by a laser at 890 nm, allowed for the detection of blood flow in the vessels. After injection to rats, an image of the cerebral vessels was succeeded in a short time; within 10 s, the vessels of the skull were perfused, indicating an efficient material for diagnosing cerebrovascular diseases. Moreover, NC uptake in the liver and spleen was observed after 24 h, thanks to the sequestration of the NCs by the cells of the endothelial reticulum [178]. Accordingly, in the work of Yu et al., cadmium-and-lead-free NCs were exploited for brain tumor diagnosis. The nucleation of the NCs took place at low temperatures, forming CuInS2/ZnS NCs, which fluoresce in the near infrared region, prepared with diphenylphosphine sulfide (SDPP) as a precursor of elemental S. These systems were able to locate a brain tumor after administration [179]. The work of Wang et al. showed the preparation of luminescent probes, consisting of ytterbium-doped NCs, emitting at 1525 nm. Usually, the systems that emit at this wavelength cause the heating of biological tissues. The aim of this work was the detection of the cerebral vascularization in the brain of a mouse through fluorinated NCs. These NCs sensitized by erbium Er3+ were brought into the aqueous phase by covering with polyacrylic acid (PAA) without affecting the strong luminescence. NCs with a core/shell structure were obtained by co-precipitation at high temperatures [180]. The work of Sojka et al. dealt with rare-earth NCs to evaluate the transport of GABA and glutamic acid in the brain. In particular, the work compared two types of hydrophilic NCs: pegylated NCs and NCs that were surface modified by the addition of -OH groups. These hydrophilic yttrium and sodium-fluoride-based NCs were doped with Eu3+ to achieve fluorescence and showed promise in replacing and overstepping the limits of the fluorophores that are often used in diagnostics. The study compared several nanoparticle systems, among which NCs resulted to be the least neurotoxic. NCs have proved to be neuroactive, able to absorb glutamic acid and GABA in the terminals, useful in the case of neurological treatments and neurosurgery [181].

#### 5.2.2. Metal-Based Nanocrystals 

A variety of quantum dots are made from heavy metals, such as cadmium/lead, which allow the imaging of specific areas. In the work of Morales-Narváez et al., cadmium–selenide/zinc sulfide quantum dots were prepared. These type of quantum dots are able to localize the ApoE protein and, based on its quantification, the progress of AD can be assessed, since abnormal levels of the ApoE isoforms can be found in such pathology. The performance of the cadmium–selenide/zinc sulfide quantum dots and Alexa 647 fluorescent dye was evaluated simultaneously by microarray and ELISA analyses, under the same conditions to form sandwich immune complexes. From the study emerged that NCs were able to reveal the different apo-lipoprotein isoforms and detect the ApoE to a greater extent than the Alexa 647 dye [182].

Perovskite NCs have recently aroused interest in diagnostics. They consist of a general formula, ABX3, in which A is a monovalent cation, B is a divalent metal cation and X is usually a halide. In particular, these systems have proven to be useful in the diagnostics of brain areas, with an optimal emission spectrum at 1700 nm [183]. In another work, KMnF3 NCs were prepared for glioma diagnosis. These metal fluoride-based NCs have high resolution and precision and a good contrast to distinguish between the labeled tissue and surrounding tissue. NCs were prepared using the “one-pot” method and then coated with albumin to improve their biocompatibility. The conjugation with albumin facilitated the passage of the NC system through the BBB. After intravenous administration (5 mg Mn/kg), a good distribution was found, mostly in the perfused organs. Evaluating the toxicity, important in the case of contrast media, a hemolysis less than 1% was revealed. The images presented in the work of Wang et al. demonstrated NCs’ capacity to detect tumor areas as very bright areas, being a good prospect for the diagnostic evaluation of cerebral tumors on an inflammatory basis [184]. 

Iron oxide NCs are also applied in brain diagnostics. In particular, these systems include magnetite and maghemite NCs coated with a stabilizer to avoid agglomeration. Their application consists of the detection of brain lesions and brain tumor or in the diagnosis of vessel infiltration following an ischemic stroke (which leads to neurodegeneration). Among these systems, two classes can be distinguished: superparamagnetic iron oxide (SPIO) and ultrasmall superparamagnetic iron oxide (USPIO) particles with dimensions below 50 nm. Their peculiar characteristic is that they are picked up by the macrophage system. Therefore, they are useful in the diagnosis of pathologies with high macrophage activity, such as in neurodegenerative diseases where the neuroinflammatory component is very high [185]. In the work of Danscher et al., it has been shown that the injection of sodium selenide or sodium selenite leads to the systemic formation of zinc–selenium NCs in the synaptic terminals of glutaminergic and GABAergic neurons. It can be used as a useful tool for brain diagnostics, but it is important that the used dose of sodium selenide injected is sufficiently high to ensure the filling of zinc-enriched synaptic vesicles (ZEN) to be detected. Larger amounts of selenide injected intracerebrally led to the increased formation of zinc–selenium molecules, which then agglomerate to form NCs. The selenium method was introduced in 1982 as a tool for zinc ion tracing in vesicular compartments, such as ZEN terminals in the CNS. With this method, it is possible to track glutaminergic and GABAergic neurons containing Zn-T3 proteins in their vesicular membrane [186].

#### 5.2.3. Nanocrystal-Based Biodevices

Synaptic devices are highly reliable devices that allow the synapses of the brain to be reproduced electrically thanks to a system that modifies its characteristics based on the strength of the electrical signals. In the work by Zhao et al., a multilayer system constituted by a layer of silicon NCs, in which subsequent layers were deposited on the top of each other by thermal evaporation, was prepared. These devices have several important synaptic functionalities, including coupled impulse facilitation (PPF), short-term transition plasticity (STP) to long-term plasticity (LTP), and time-dependent plasticity to peak (STDP). The authors aimed to obtain a device that allowed the synapses to resume their function through the conduction of the impulse between the presynaptic axon and the postsynaptic dendritic terminal. Therefore, the light-emitting diodes of silicon NCs showed their ability to exhibit a series of important synaptic functionalities, mimicking biological synapses [187]. Finally, the development of a biosensor for the detection of adrenaline in biological fluids is discussed. Adrenaline is a hormone synthesized by the adrenal medulla, which acts as a neurotransmitter for the sympathetic nervous system. It is involved in neurodegenerative diseases, such as Parkinson’s. It proved to be an excellent method for medical brain diagnostics, as the biosensor was tested on the zebrafish brain. Among the constituents of the biosensor, cellulose NCs can be mentioned. The dosimetric biosensor reproduced a signal based on the amount of adrenaline detected. It showed a good selectivity towards adrenaline and could be used in the diagnosis of neurodegenerative diseases [188]. 

## 6. Market and Patents of NCs for Brain Delivery

During the last two decades, the FDA approved different nano-formulations, including drug NCs, liposomes and polymeric NPs [189]. Multiple challenges can impact the drug development pipeline and influence the regulatory process, especially if “nano” drugs intended to be delivered to the brain are considered. This is due to the potential cerebral nanotoxicity induced by particle size, morphology and charge that can trigger inflammatory phenomena, apoptosis, and oxidative stress. Therefore, despite the numerous advances in this field, only a few patents concerning nanotechnological formulations for the treatment of neurodegenerative diseases are currently available [190]. Currently, several NC formulations are under clinical trial as reported in recent reviews [191,192,193]. NCs and/or nanosuspensions have aroused great interest in the last twenty years. The preparation method, easily scalable at an industrial level, combined with the advantages of the final product led to the revaluation of this formulation for its placing in the market. However, before being placed in the market, the nanosuspensions have often been transformed into different final pharmaceutical forms. Drying and freeze drying, followed by their transformation into tablets, are the main “finished” products. In fact, one of the most exploited technological strategies is to dry the nanosuspension and transform it into an easily disintegrating oral pharmaceutical form. The first NC-based product marketed by the FDA was Rapamune^®^, which consists of a tablet form providing easier administration and storage than the currently marketed Rapamune^®^ oral solution. Developed by Elan’s Elan Pharmaceutical Technologies (EPT) division, the NanoCrystal^®^ technology enabled the efficient delivery of poor water-soluble drugs [194]. Emend^®^ was approved by the FDA in 2003 [195]. It is composed of aprepitant, a central antiemetic drug, for the prevention of nausea and vomiting induced by chemotherapy, characterized by a very low oral bioavailability and water solubility. Emend^®^, produced by Elan’s NanoCrystal technology, improved drug water solubility and absorption in the gastrointestinal tract. Moreover, studies showed that that treatment using Emend^®^ in chemotherapy with cisplatin, doxorubicin and cyclophosphamide did not result in significant pharmacokinetic interactions. Furthermore, the combination of Emend^®^ with dexamethasone and ondansetron in the treatment of nausea and vomiting induced by chemotherapy managed to contain acute, but also delayed, emesis [196].

Aristada Initio^®^, Invega Sustenna^®^ and Xeplion^®^ can be grouped in the same class of antipsychotics: LAI (long-acting injection) drugs. LAI is a class of “depot” antipsychotics that represented a “revolution” in the treatment of schizophrenic patients, considering that the main problem related to schizophrenia is given by the long-term therapy, since the repeated administration regimen leads to poor patient compliance and adherence to therapy (mainly oral). Currently, the commercially available pharmaceutical form of LAI is an intramuscular injection. Among LAI, paliperidone palmitate, olanzapine pamoate and aripiprazole prolonged release and risperidone prolonged release can be mentioned [197,198]. Aristada^®^ and Aristada Initio^®^ (aripiprazole lauroxil NCs dispersion) are two FDA-approved nanosuspensions for intramuscular injection for the treatment of schizophrenia. Aristada Initio^®^ is a colloidal dispersion of NCs approved by the FDA on 2 July 2018, as a one-time injection. Conversely, Aristada^®^ is an extended-release injectable suspension and was approved in October 2015. Both are depot formulations, whose therapeutic protocol provides an initial treatment with Aristada Initio^®^ in combination with the oral aripiprazole (Abilify^®^) to start Aristada^®^ (aripiprazole lauroxil) treatment. Interestingly, pharmacokinetic studies have shown that a one-day use of this injection is comparable to the conventional therapy consisting of one injection of aripiprazole (Aristada^®^) and 21 days of oral aripiprazole in tablets. It is a 675 mg single dose nanosuspension that has a sustained initial drug release rate due to its small particle size. The strategy of decreasing the dimensions to the order of nanometers, compared to the formulation of Aristada^®^, allows for improved release kinetics and an improved bioavailability of the active ingredient, while maintaining the same prodrug as a base. Conventional therapy is thus replaced with single dose of Aristada Initio^®^ before a single 30 mg dose of oral aripiprazole together with an injection of Aristada^®^. The bioavailability of Aristada Initio^®^ rises to 100% with a half-life of 15–18 days [199,200]. Phase I pharmacokinetic and safety studies demonstrated a good bioavailability profile, assessed by the evaluation of log-transformed PK parameters of exposure (C_max_, AUC_last_, and AUC_∞_) with an injection of aripiprazol lauroxil NC dispersion in both the deltoid and the gluteus, demonstrating both injection sites are safe and efficient [201]. From the early 1990s to the present date, the technologies used to produce NCs at an industrial scale are wet ball milling (WBM), also known as NanoCrystal^®^ technology, and HPH to decrease particle size. Using the WBM technology, the first parenteral pharmaceutical product was produced. It was based on paliperidone palmitate NCs. To date, two formulations are available on the market based on paliperidone palmitate: Invega Sustenna^®^ and Xeplion^®^. Both consist of pre-filled syringes for intramuscular administration [202]. Paliperidone palmitate is the ester of paliperidone, which is normally insoluble in water. Drug nanonization and conversion into nanosuspension allowed to obtain the Invega Sustenna^®^ product, allowing for the greater dissolution of the active ingredient and a better bioavailability. The use of low concentrations of a stabilizer (polysorbate 20 and 80) guarantees the safety of the product for parental administration. It is a slow-release formulation with a patient-friendly treatment regimen consisting of a single high-dose initiation followed by a maintenance dose [203]. Xeplion^®^ is composed by the same prodrug (Invega^®^ for the U.S. market; Xeplion^®^ for the E.U. one). It is a suspension of paliperidone palmitate NCs in an aqueous buffer solution (pH 7.1) for prolonged drug release after injection [204]. The drug is safe and well tolerated. In addition to the better patient compliance and adherence to the therapy compared to the oral antipsychotic, the EMA reported a tolerability comparable to the oral dosage form, except for the usual reactions at the administration site, typical of intramuscular injections [205,206]. The onset of the clinical response of paliperidone palmitate (Invega Sustenna^®^, Xeplion^®^) is 8 days after the first day of dosage. The initial E.U. dosing regimen of intramuscular paliperidone palmitate is a 150 mg single dose on the first day and then 100 mg after one week on day 8, both intramuscular injections into the deltoid [207]. 

Among the FDA-approved products based on NCs, there are two psychostimulants, in which the bioavailability of methylphenidate is improved. The two products are Ritalin^®^, used in attention deficit hyperactivity disorder (ADHD), and Focalin^®^, which is a psychostimulant also used in ADHD [208,209]. Both products are produced by the media milling technology [210]. As shown in Table 3, Zyprexa Relprevv^®^ is another formulation approved by the FDA in 2010 based on NCs of the olanzapine pamoate prodrug, belonging to the LAI class. The use of Zyprexa Relprevv^®^ overcomes the serious and typical side effects related to the oral drug formulation, such as the post-injection delirium sedation syndrome (PDSS) [211]. NC technology is a very promising technique due to its ease of production and attractive pharmaco-economic values. The global NC market is predicted to generate a revenue of USD 233.16 million in 2027, which was USD 82.5 million in 2020 (https://www.credenceresearch.com/toc/nanocrystal-market/table-of-content accessed on 28 January 2022). The progress of the NC market depends entirely on the assurance of patients, commercial success, and safety of nanomedicines. To overcome this challenge, the future market needs a greater public awareness of the benefits, risks and safety concerns related to nano-pharmaceuticals.

## 7. Challenges and Perspectives

The importance of NCs has grown over the past decade with new nanomedicine products entering the market and approved for clinical purposes by the FDA. The global NC market is projected to reach many millions by 2028 compared to 2021, with an unexpected compound annual growth rate (CAGR) during the same period [212]. In particular, reports highlight the growth of the market for cellulose and silicon-based NCs (not discussed in this review, as they are largely detailed in recent reviews). Among the wide applications of these nano-pharmaceuticals, brain delivery represents an urgent need, and it has not yet become a widely explored field. Furthermore, in the field of nanomedicine, there is a great interest on innovation, but the applicability of complex and factitiously designed pharmaceutics is often unfeasible. If, on the one hand, structural complexity can enhance the success of the developed nanosystem, on the other hand, problems related to toxicity and regulatory affairs can arise. NCs, thanks to their structural simplicity and scalable manufacturing processes, have the potential to revolutionize the therapeutic and diagnostic sectors and their application is expected to emerge, thus deserving considerable attention. 

## 8. Conclusions

The clinical application of many active molecules remains a challenge due to the poor water solubility and bioavailability issues of ~90% of natural and synthetic molecules in the development pipeline. In order to overcome this critical issue, the design of drug NCs, with reduced dimensions in the nanometric order, can be exploited to promote a faster drug dissolution rate, greater solubility and bioavailability. The simplicity of NC composition and the possibility to transform these nano-pharmaceuticals into different pharmaceutical final forms results in a growing interest of the NC technology, especially for drug delivery to the brain. Drug delivery to the brain is still challenging nowadays, due to the presence of biological barriers that hinder the passage of foreign molecules. New strategies to improve drug transfer to the brain are under investigation. Nose-to-brain delivery is emerging as an alternative non-invasive method to directly transfer drug molecules inside the brain. Drug NCs could be considered promising candidates for intranasal administration to CNS areas since the high drug loading and the possibility to maintain a very potent therapeutic action is particularly useful in the treatment of neurodegenerative diseases. The application of drug NCs for neurological diseases (Parkinson’s, AD, psychosis, etc.) is currently under deep investigation by researchers as revealed by the recent and growing studies in this field, even if the application of nose-to-brain delivery has yet to be explored in depth. In addition to the therapeutic field, NCs are being widely studied even for diagnostic application since inorganic materials can be used to obtain fluorescent semiconductor NCs for high-resolution cell imaging, as well as for tumor targeting. Overall, NC systems are gaining great attention as revealed by the clinical effectiveness of NCs, which is demonstrated by the increasing number of FDA-approved NC products on the market for the treatment of neurodegenerative diseases, which is expected to grow in the coming future.

## Figures and Tables

**Table 1:** NCs for different routes of administration.

Administration Routes	Stabilizers	Method of Preparation	Final Pharmaceutical Dosage Form	Particle Size	Drug	BCS Class	Disease	Aim of the Work	Refs.
ORAL	Methylcellulose (MC) and HPβCD	Bead mill method	Suspension of solid nanoparticles	20–180 nm	Meloxicam	II	Rheumatoid arthritis	Reduce GI ulcerogenic responses	[29]
	Soluplus	Precipitation–ultrasonication method	Capsules	244 nm	Cinacalcet hydrochloride	IV	Chronic kidney disease	Improve absorption Reduce the food effect	[30]
	Sodium dodecyl sulfate (SDS) and Hydroxypropyl methyl cellulose (HPMC)	Wet media milling	Oral Strip Films	285 nm	Fenofibrate	II	Reduce blood fat levels	Enhanced oral bioavailability	[31]
	poloxamer F127, HPMC-E5, sodium deoxycholate	Microprecipitation and high pressure homogenization	Solid dispersion	833.3 nm	Nimodipine	II	Calcium channel blocker	Increase the dissolution rate	[32]
	SDS and Alpha-tocopherol succinate (VES)	Antisolvent precipitation–ultrasonication	Nanosuspension	212 nm	Carvedilol	II	Non-selective β-blocking agent	Comparison with market formulations	[33]
	HPMC	High pressure homogenization method (HPH)	Lyophilized powder	From 1875.6 nm to 525.8 nm	Puerarin	IV	Cardiovascular diseases	Investigation of the influence of particle size on oral pharmacokinetics	[36]
	poloxamer 407 (F127),poloxamer 188 (F68), hydroxypropyl methyl cellulose E5 (HPMC-E5) and sodiumdeoxycholate (NaDC)	Wet media milling	Nanosuspension	From 360.2 nm to 376.9 nm	Spironolactone	II	Model drug (diuretic)	Investigation of the influence of stabilizers in particle size	[37]
	PVP, ethyl cellulose, HPMC, PVA, Pluronic F127 and Eudragit (EUD)	Low-energy antisolvent precipitation method	Nanosuspension	From 130 nm to 1200 nm	Domperidone	II	Antiemetic	Selection of suitable polymers to improve the bioavailability	[38]
	Pectin, SDS, HPMCAS, Lecithin, HACC	Wet grinding	Nanocrystals	From 209 nm to 438.23 nm	Bufadienolides	/	Model drug	Exploration of the efficiency of penetrating through the mucus layer and transporting intestinal epithelial cells	[39]
	/	High pressure homogenization method	Nanocrystal self-stabilized Pickering emulsion	390.9	Puerarin	IV	Cardiovascular diseases	Improve the oral bioavailability of puerarin	[41]
	Methylcellulose and 2-Hydroxypropyl-_-cyclodextrin	Bead mill method	Tablet	140 nm	Irbesartan	II	Antihypertensive	Investigation of the effects on blood pressure	[42,43]
	Pluronic F127	Wet milling	Lyophilized powder	274.3	Naringenin	II	Anti-inflammatory	Rheumatoid arthritis treatment	[44]
OCULAR	cetylpyridinium chloride and benzalkonium chloride	Wet bead milling	Nanosuspension	200–250 nm	Dexamethasone acetate	III	Corticosteroid	Increase retention time and final bioavalability	[49]
	Pluronic F-127 and methylcellulose	Bead mill	In situ gel	50–170 nm	Indomethacin	II	NSAIDs	Enhanced absorption	[50]
PARENTERAL	Hydroxypropyl methylcellulose and PVP	Microfluidic technology	Nanosuspension	210–280 nm	Hydrocortisone	II	Corticosteroid	Studies of microfluidic techniques	[27]
	poloxamer 188	Anti-solvent/ nanoprecipitation method in combination with homogenization	Fluorescent nanosuspension	300 nm	Curcumin	IV	Antioxidant model drug	Drug tracking	[57]
	Tween 80, VolpoL4, Plasdone, Poloxamer 188, PVP	High pressure homogenization technique	Nanosuspension	457.6 nm	Nevirapine	II	HIV infection	Create a viral reservoir targeting	[58]
	Pluronic F127	Wet milling	Nanosuspension	400 nm	Paclitaxel	IV	Chemoterapic	Hyperthermic intraperitoneal chemotherapy (HIPEC)	[63]
	Cremophor ELP	Anti-solvent and temperature-induced crystallization	In situ crosslinkable hydrogel depot	258.0 nm	Paclitaxel	IV	Chemoterapic	Intraperitoneal chemotherapy of ovarian cancer	[64]
PULMONARY	D-α-tocopheryl polyethylene glycol 1000 succinate (TPGS)	Nanoprecipitation with sonication	Dry powders for inhalation	823–931 nm	C109	/	Cystic fibrosis (CF)	Development of mucus-penetrating formulation	[66]
	poloxamer 188	Wet milling method	Nanosuspension	246.16 nm	Curcumin	IV	Model drug	Studied the size effect on pulmonary absorption	[68]
	Tween 80	Wet milling combined with the spray drying method	Spray-dried powders for inhalation	924 nm	Curcumin	IV	Model drug	Dry powders for inhalation (DPI)	[70]
DERMAL	Poloxamer 407	Wet bead milling	Nanosuspension	260–290 nm.	Dexamethasone	III	Corticosteroid	Inflammatory skin diseases	[74]
	Plantacare^®^ 2000 UP	High pressure homogenization (HPH)	Nanosuspension	429 nm	Lutein	II	Antioxidant and anti-free radical	Enhance penetration into the skin	[75]
	Tween_ 80, TPGS, Lutrol_ F68, Plantacare_ 810 or Plantacare_ 1200	SmartCrystals_ technology (combines bead milling and subsequent high-pressure homogenization at relatively low pressure)	Nanosuspension and gel	295 nm and 203 nm	Quercetin	II	Antioxidant and antiradicals	Evaluation of antioxidant activity and cellular safety	[76]
	Poloxamer 188, Inutec SP1, Tween 80 and Plantacare 2000	High-pressure homogenization	Nanosuspension	300 nm	Hesperetin	II	Anti-aging	Elucidate which type of stabilization proves most effective	[77]
	HPMC E15 or MC	Antisolvent precipitation technique	Wafers	In the range of 300 nm	Diosmin	IV	Anti-inflammatory, antioxidant, antiulcer, anticancer, antiatherogenic, hepatoprotective, and neuroprotective	Treatment of diabetic ulcer	[78]
	TPGS (d-_-tocopherol polyethylene glycol 1000 succinate)	Small-scale bead milled curcumin nanosuspension	Nanosuspension	250 nm	Curcumin	IV	Model drug	Hair follicle accumulation	[79]
	Tween 80 or P188	Top-down media milling method	Nanosuspension after micro-needle roller treatment	264 and 279 nm	Diclofenac	II	NSAIDs	Improve transdermal penetration combining the use of DCF nanosuspensions with a microneedle roller	[81]
INTRANASAL	Tween 80 or F68 plusPVP-K25 Second study only F68	Precipitation– ultrasonication method	Nanosuspension	Below 500 nm (first study) and 300 nm (second study)	Loratadine	II	Antihistamine drug	(Local) allergic conditions, such as rhinitis, urticaria and atopic dermatitis	[85,86]
	Tween 80, Solutol HS 15, Span 40, PVP K-30, poloxamer 407 and poloxamer 188, stearic acid, oleic acid	Modified precipitation– ultrasonication method	In Situ Gelling Nasal Spray	190 nm	Carvedilol	II	Non-selective β-blocking agent	Immprove bioavailability than the oral forms	[87]
	polyvinylpyrrolidone (PVP) or semi-crystalline polyethylene glycol (PEG)	Co-grinding process	Dry powder	174 nm	Meloxicam	II	NSAIDs	Systemic nasal delivery	[88]
	Poloxamer 407 and Poloxamer 188	Nanoprecipitation– ultrasonication method	Mucoadhesive Nanosuspension Nasal Spray	/	Ivermectin	II	Anti-parasitic agent	Reduce upper respiratory symptoms of mild COVID-19	[89]
	Tween 80	High pressure homogenization (HPH)	Nasal spray	Less than 250 nm	Fluticasone propionate	II	Corticosteroid	Nasal spray formulation for rhinitis therapy	[91]
	polyvinylpyrrolidone (PVP K90)	Top-down nanocrystal technology	Hydrogel	101.47 nm	Armodafinil	II	Used in clinical practice to maintain cognition and wakefulness in patients suffering from sleep deprivation	Sleep deprivation	[92]
	SoluplusVR, a-TPGS, or Poloxamer-188	Anti-solvent precipitation process followed by the probe sonication method	Powder	223.16 nm	Zaleplon	II	Non-benzodiazepine hypnotics	Insomnia, improving zaleplon (ZAP) performance and escape first-pass metabolism	[93]
	Chitosan	Ionic crosslinking method	Nanosuspension	150–200 nm	Donepezil	II	Cholinesterase inhibitor	Nose-to-brain Alzheimer’s disease	[104]
	Pluronic F-127, HPMC, soya lecithin	Onoprecipitation (SP) and combination technique (high pressure homogenization preceded by precipitation)	Nanosuspension	519.26 nm & 330.2 nm	Zotepine	II	Anti-psychotic	Psychosis, increased brain targeting with lower doses	[105]
	/	Anti-solvent precipitation method	In situ gel	Near 241 nm	Resveratrol	II	Antioxidant	Neurodegenerative diseases via the suppression of the formation and aggregation of amyloid peptides associated with Alzheimer’s dis-ease owing to its potent antioxidant actions	[108]
	TPGS	Antisolvent precipitation method	Nanosuspension	139.6 nm	Paeoniflorin	III	Anti-Parkinson	Evade BBB to increase the brain concentration of PA	[109]
	Tween 80	High-pressure homogenization method	In situ gel	527 ± 1 nm	Breviscapine	IV	Flavonoids Chinese medicine	Nose-to-brain model drug	[110]
	TPGS	Coupling homogenization and spray-drying technology	In situ nanogel	179.21	Harmine	II	Model drug	Alzheimer’s disease study using a carbohydrate polymer deacetylated gellan gum (DGG)	[111]
	PVP, Tween 80, F68	Combination of precipitation and ultrasonication (sonoprecipitation)	Nanosuspension	328.7 nm	Curcumin	IV	Model drug	Model drug to evaluate delivery	[112]

**Table 2:** NCs for different neurodegenerative pathologies.

Neurological Disease	Drug	Administration Route	In Vitro Studies	In Vivo Studies	Comparison with Market Product	Refs.
PARKINSONS DISEASE	Schisanterin	Oral	Cellular transport of MDCK cells; intracellular integrity by the FRET technique; real-time drug release monitoring	Plasma and brain pharmacokinetic studies in rats; neuroprotective bioassays on zebrafish and cell culture models of PD	Not applicable	[138]
	Ginkolide B	Oral	Cellular uptake and transport of GB-NCs in MDCK cell monolayers; MTT assay for the cytotoxicity of GBNCs against SH-SY5Y cells	In vivo toxicity assays (zebrafish); in vivo pharmacokinetics (rat model); neuroprotective assay	Not applicable	[139]
	Resveratrol	Oral	Assessment of RES-NCs cellular uptake and transport (MDCK cell); assessment of RES-NCs protection against MPP + mediated cell death (SH-SY5Y cells)	Evaluation of in vivo RES-NCs toxicity (zebrafish embryon); pharmacokinetic analysis of RES-NCs in rats; neuroprotective effects; Akt/Gsk3 β signaling pathway in PD model mice	Not applicable	[140]
	Quercetin	Oral	/	Neuroprotective effects of quercetin nanocrystals on 6-hydroxydopamine (6-OHDA)-induced Parkinson-like model in male rats; determination of superoxide dismutase activity; determination of catalase activity and total glutathione content after NCs administration; determination of malondialdehyde	Not applicable	[141]
	Puerarin	Oral	Cellular uptake and permeation of PU-NCs through MDCK cell monolayers; effects of PU-NCs on MPP + Induced Cytotoxicity in SH-SY5Y Cells	In vivo toxicity analysis (zebrafish); intestinal absorption and brain uptake of PU-NCs investigated by the FRET imaging technique; pharmacokinetic analysis of PU-NCs in rats	Not applicable	[144]
PSYCHOSIS	Aripiprazole	Buccal	In vitro drug release	Ex vivo permeation studies	/	[148]
	Risperidone	Oral	In vitro drug release	/		[149]
	Ziprasidone	Oral	Cell viability and permeability in Caco-2 cells	/	Compared with the marketed Zeldox^®^ capsule	[150]
	Risperidone	Oral	In vitro dissolution studies of the prepared formulae	In vivo drug absorption study (rabbits)	Compared with the marketed tablets (Risperidal^®^)	[151]
ALZHEIMER’S DISEASE	Calpain inhibitor I and SNJ-1945	/	/	/		[155]
	Donepezil	Intramuscular injection	Cell viability study on the murine fibroblast cell line 3T3 NH	Pharmacokinetic study; in vivo safety evaluation; in vivo pharmacodynamic study in intracerebroventricular (ICV) streptozotocin (STZ)-induced memory impairment model; brain acetyl choline esterase (AChE) activity	/	[156]
CEREBRAL ISCHEMIA	PX-18	Intraperitoneal injection	/	Neuroprotective assay	/	[157]
BRAIN INFECTION	Nevirapine	Intravenous	In vitro protein adsorption; cytotoxicity studies	Uptake, pharmacokinetic and biodistribution studies	/	[58]
	Curcumin	Intravenous	/	Pharmacokinetics study (rats); Tissue distribution	/	[162]
	Atovaquone	Oral	Cellular uptake in Immortalized b.End3 mouse brain capillary endothelial cells (in vitro blood barrier model)	Uptake in murine model	/	[163]
	Atovaquone	Oral	Cytotoxicity on murine macrophage (J774A.1), murine intestinal epithelial (MODE-K), and murine brain endothelial (bEnd3) cell lines	Therapeutic efficacy of atovaquone in a murine model of acute acquired toxoplasmosis: atovaquone concentrations in serum and organs, and antiparasitic effect of atovaquone	Compared to Wellvone^®^	[164]
	Amphotericin B		In vitro assay for drug efficacy against B. mandrillaris amebas	Tissue distribution, (immunostaining, histological assay, HPLC analysis)	Compared to Ambisome^®^	[165]
	Docetaxel	Intravenous	In vitro targeting and efficacy	In vivo biodistribution and antitumor efficacy on an orthotropic glioma model; in vivo biodistribution and antitumor efficacy on a subcutaneous tumor model	/	[167]
	20(S)-protopanaxadiol	Oral	Transport experiments in MDCK cell monolayers	Pharmacokinetic study in rats	/	[168]
	Docetaxel	Intravenous	In vitro drug release studies	In vivo brain distribution study in rats	Compared to Docel^®^	[169]
DEMYELINATING PATHOLOGIES	CNM-Au8	Oral	In vitro differentiation of OPCs with treatment of nanocrystalline gold; in vitro RNAseq expression study; in vitro quantitation of NAD+ and NADH; in vitro quantitation of ATP	Remyelination assay by nanocrystalline gold using the in vivo cuprizone model of demyelination	/	[172]
	Curcumin	Intraperitoneal	In vitro evaluation of total ROS and mitochondrial superoxide in Schwann cells (SCs); in vitro evaluation of mitochondrial membrane potential (ΔΨm) in SCs	Investigatation of the therapeutic potential in CMT1A transgenic rats	/	[173]

**Table 3:** Nanocrystal/nanosuspension-based products approved by the FDA for neurological disorders.

Product	Drug	Pathology/Role	Pharmaceutical Forms	Composition	Company	Administration Route	Year
Emend^®^	Aprepitant	Central anti-emetic	Tablets	Aprepitant, Sucrose Microcrystalline cellulose (E 460), Hydroxypropylcellulose (E 463), Sodium laurilsulfate, Capsule shell	Merck & Co., Inc., Kenilworth, NJ, USA	Oral	2003
Aripiprazole Lauroxil Nanocrystals (Aristada Initio)^®^	Aripriprazole Lauroxil	Schizophrenia	Injection (IM)	Sorbitan monolaurate, Polysorbate 20, Sodium citrate dihydrate, Sodium chloride, Monobasic sodium phosphate dihydrate, Dibasic sodium phosphate anhydrous, Water for injection	Alkermes, Inc., Waltham, MA, USA	Intramuscolar	2015
Invega Sustenna^®^/Xeplion^®^	Paliperidone Palmitate	Schizophrenia	Injection (IM)	Paliperidone palmitate, Citric acid, Monohydrate, Disodium hydrogen phosphate anhydrous, Sodium dihydrogen phosphate monohydrate, Sodium hydroxide, Polyethyleneglycol 4000, Polysorbate 20, Water for injection	Janssen, Belse, Belgium	Parenteral/Intramuscolar	2009
Ritalin LA^®^	Methylphenidate HCl	Psychostimulant/ADHD	Tablets	Methylphenidate HCl, Ammonium methacrylate copolymer, Black iron oxide (10 and 40 mg capsules only), Gelatin, Methacrylic acid copolymer, Polyethylene glycol, Red iron oxide (10 and 40 mg capsules only), Sugar spheres, Talc, Titanium dioxide, Triethyl citrate, Yellow iron oxide (10, 30, and 40 mg capsules only)	Novartis, Basel, Switzerland	Oral	2002
Focalin XR^®^	Dexmethylphenidate HCl	Psychostimulant/ADHD	Tablets	Dexmethylphenidate HCl, Ammonium methacrylate copolymer, FD&C Blue #2 (5 mg strength), FDA/E172, Yellow iron oxide (10 mg strength), Gelatin, Ink Tan SW-8010, Methacrylic acid copolymer, Polyethylene glycol, Sugar spheres, Talc, Titanium dioxide, Triethyl citrate	Novartis, East Hanover, NJ, USA	Oral	2005
Zyprexa Relprevv^®^	Olanzapine pamoate	Schizophrenia	Intramuscular injection	Olanzapine pamoate, Carboxymethylcellulose sodium, Mannitol, Polysorbate 80, Sodium hydroxide and/or hydrochloric acid for pH adjustment, Water for injection	Eli Lilly, Indianapolis, IN, USA	Parenteral	2010

## Data Availability

Not applicable.
